# Prognostic and biological function value of OSBPL3 in colorectal cancer analyzed by multi-omic data analysis

**DOI:** 10.1186/s12876-023-02824-1

**Published:** 2023-08-07

**Authors:** Chengxing Wang, Yaoming He, Yu He, Weijun Liang, Chaorong Zhou, Meimei Wu, Zijie Meng, Wanglin Li, Jie Cao

**Affiliations:** 1 The First Affiliated Hospital, Jinan University, Guangzhou, 529000 Guangdong China; 2 Department of Gastrointestinal Surgery, Jiangmen Central Hospital, Jiangmen, 529000 Guangdong China; 3 National Drug Clinical Trial Institution, Jiangmen Central Hospital, Jiangmen, 529000 Guangdong China; 4 Clinical Experimental Center, Jiangmen Key Laboratory of Clinical Biobanks and Translational Research, Jiangmen Central Hospital, Jiangmen, 529000 Guangdong China

**Keywords:** OSBPL3, Colorectal cancer, Bioinformatics, Prognosis, Histological analysis

## Abstract

**Background:**

Colorectal cancer (CRC) is one of the most common malignancies in the world. This study proposes to reveal prognostic biomarkers for the prognosis and treatment of CRC patients.

**Methods:**

Differential analysis of OSBPL3 was performed in pan-cancer, and the correlation between clinical stage and OSBPL3 was analyzed. Multiple omics analysis was used to compare the relationship between survival of patients and copy number variation, single nucleotide variant, and methylation status. Survival differences between high and low OSBPL3 expression groups were analyzed. Differentially expressed genes (DEGs) between high and low OSBPL3 expression groups were obtained, and functional enrichment analysis was implemented. Correlations between immune cells and OSBPL3 was analyzed. Drug sensitivity between the two OSBPL3 expression groups was compared. Moreover, the expression of OSBPL3 was verified by immunohistochemistry and real-time quantitative PCR.

**Results:**

OSBPL3 was differentially expressed in 13 tumors and had some correlations with T and N stages. OSBPL3 expression was regulated by methylation and higher OSBPL3 expression was associated with poorer prognosis in CRC. 128 DEGs were obtained and they were mainly involved in signaling receptor activator activity, aspartate and glutamate metabolism. T cell gamma delta and T cell follicular helper were significantly different in the high and low OSBPL3 expression groups. Moreover, OSBPL3 showed negative correlations with multiple drugs. OSBPL3 was significantly upregulated in CRC samples compared to normal samples.

**Conclusions:**

A comprehensive analysis demonstrated that OSBPL3 had potential prognostic value, and guiding significance for CRC chemotherapeutic.

**Supplementary Information:**

The online version contains supplementary material available at 10.1186/s12876-023-02824-1.

## Introduction

Colorectal cancer (CRC) is the third common cancer in the world but ranks second in terms of mortality worldwide, and its mortality rate is 9.4%. CRC caused 1.93 million new diagnosed cases and 935,000 deaths in the whole world in 2020 [1]. Surgery is an important treatment for CRC, but postoperative recurrence and metastasis lead to poor prognosis. Chemotherapy and radiotherapy are more common in the treatment of advanced CRC. However, they have many side effects such as neutropenia, diarrhea, vomiting and radiation enteritis due to non-specific cytotoxicity [2, 3]. Immunotherapy can selectively target and kill tumor cells without toxic effects on normal cells, and it can avoid the toxic side effects and immunity decline caused by traditional radiotherapy and chemotherapy. Therefore, immunotherapy has become a new alternative in advanced CRC treatment, especially for those with chemotherapy resistance, but the prognosis of different patients with immunotherapy is uncertain due to genetic heterogeneity.

Oxysterol-binding protein like protein 3 (OSBPL3) is a group of intracellular lipid receptors [4], that has a C-terminal OSBP domain and an N-terminal pleckstrin homology (PH) domain [5]. It is mainly expressed in endoplasmic reticulum and plasma membrane of human cells, and which was confirmed to participate in physiological processes such as cell adhesion and lipid signaling [6–8]. In addition, transcriptome analyses showed that OSBPL3 is associated with tumorigenesis including colorectal [9], pancreatic ductal [10], pancreatic head [11], gastric cancer and metastatic breast cancer [12, 13]. More importantly, OSBPL3 has a significant effect on CRC progression through activation of immune mechanism [14]. The strong correlation between OSBPL3 and CRC predicts that OSBPL3 may be a potential prognostic biomarker of CRC treatment. Therefore, identification of OSBPL3 in CRC is crucial to determine the prognosis of immunotherapy in CRC patients.

In this study, we aim to assess prognostic significance of OSBPL3 in the treatment of CRC by using different bioinformatics analysis databases and carried out clinical case validation. It contributes to reveal the role of OSBPL3 in immunoregulation and detecting chemotherapy of CRC. We present the following article in accordance with the TRIPOD reporting checklist.

## Methods

### Data source

The transcriptomic data (Tumor = 616, Normal = 51), copy number variation (CNV) data (Tumor = 616, No normal samples), single nucleotide variation (SNV) data (Tumor = 381, No normal samples), methylation data (Tumor = 309, Normal = 38), and clinical data of CRC were downloaded from The Cancer Genome Atlas (TCGA) database (https://portal.gdc.cancer.gov/). After removing the samples with no survival status and survival time from the clinical data, a total of 612 CRC patients were obtained for subsequent analysis. In addition, the transcriptomic data and survival data of 233 CRC samples were downloaded from the GSE17538 dataset in the Gene Expression Omnibus (GEO) database (https://www.ncbi.nlm.nih.gov/geo/) to validate the survival of patients. The survival data contained overall survival (OS), progression free survival (PFS), disease free survival (DFS), and disease special survival (DSS).

### Subjects and specimens

This study included 100 CRC patients confirmed by pathology in Jiangmen Central Hospital from 2016 to 2017. Colorectal tissue was collected by colonoscopy or surgery and stored in the Clinical Experimental Center, Jiangmen Key Laboratory of Clinical Biobanks and Translational Research affiliated to Jiangmen Central Hospital. There were 40 males and 60 females. Ages ranged from 20 to 75, with an average age of 56 ± 8 years. The end time point of follow-up was June 2022 or the time of patient death. The death of a patient that was not tumor-related was classified as a loss of visit. The main outcome indicator were overall survival (OS) and progression free survival (PFS). This study was approved and supervised by the ethics committee of Jiangmen Central Hospital (decision no. JXY2022107). All subjects provided signed informed consent.

### OSBPL3 expression pattern analysis

First, the expression data of oxysterol binding protein like 3 (OSBPL3) in tumor tissues and normal tissues of 15 cancers (BLCA, BRCA, CESC, COAD, ESCA, HNSC, KIRC, LIHC, LUAD, LUSC, PAAD, PRAD, READ, STAD, and THCA) were obtained from the pan-cancer database of GDC (https://gdc.cancer.gov/about-data/publications/pancanatlas). Then, the expression of OSBPL3 was compared between tumor and normal tissues of each cancer by wilcoxcon test, and the results were visualized by drawing box line plot. The combined data of COAD and READ was used to compare the expression of OSBPL3 between tumor and normal samples. Finally, the expression of OSBPL3 was compared in different clinical stage subgroups.

### Multiple omics analysis of OSBPL3 in CRC

The SNV data and CNV data of OSBPL3 were extracted to compare the OS of patients. Methylation sites were annotated using the ChAMP package (version2.20.1, RRID:SCR_012891) [15]. The differences of methylation sites between CRC and normal tissues were analyzed (*P* < 0.05, |deltaβ|> 0.1), and the correlation analysis was performed between the differentially expressed methylation sites and OSBPL3. Then, the survival correlation analysis was performed between methylation sites and patient. The patients were divided into two groups according to the optimal threshold calculated by survminer, and the OS analysis was performed between the two groups.

### Prognostic analysis of OSBPL3

OSBPL3 expression data was extracted and merged with survival times and survival status (OS, PFS, DFS, and DSS) of patients. The optimal threshold was identified using the survminer package (version 0.4.9, RRID:SCR_021094), and the patients were divided into high and low OSBPL3 expression groups according to the threshold. The survival curve was plotted using the survival package (version 3.2–11, RRID:SCR_021137) to compare the survival of two groups. Finally, the GSE17538 dataset and self-test data were used to validate the survival of patients.

### Independent prognostic analysis of OSBPL3

First, the chi-square test was used to compare the difference between OSBPL3 high and low OSBPL3 expression groups in different clinical subgroups. Then, stratified survival analysis was performed for clinical subgroups with significant differences. The independent prognostic analysis was performed by univariate and multivariate Cox regression analyses. A nomogram was constructed using rms (version 6.2–0, RRID:SCR_007415) package to predict the survival of CRC patients, and calibration curve was drawn to assess the accuracy of the prediction. Finally, we performed independent prognostic analysis of OSBPL3 and other clinical information.

### Functional enrichment analysis of differentially expressed genes (DEGs)

The DEGs between high and low OSBPL3 expression groups were obtained by limma package (RRID:SCR_010943) (*P* < 0.05, |logFC|> 0.5) [16]. Ggplot2 (version3.3.6, RRID:SCR 014601) and pheatmap package (version1.0.12, RRID:SCR_016418) were used to draw volcano plot and heat map [17, 18]. Gene Ontology (GO) and Kyoto Encyclopedia of Genes and Genomes (KEGG) [19–21] functional enrichment analyses were performed using the clusterProfile package (version 3.18.1, RRID:SCR_016884). Functional prediction of DEGs was performed using the Metascape online database (https://metascape.org/gp/index.html#/main/step1). Finally, we performed KEGG enrichment analysis by screening DEGs at adj.p < 0.05 to mine more pathways.

### Immune infiltration analysis

Cell Type Identification by Estimating Relative Subsets of RNA Transcripts (CIBERSORT) algorithm was used to analyze the immune cell infiltration between the high and low OSBPL3 expression groups [22]. The proportion of 22 immune cells in CRC samples (N = 612) was calculated. The differences of immune cells between high and low OSBPL3 expression groups were compared by wilcoxcon test. Spearman correlation analysis was performed in OSBPL3 and immune cells, ggplot2 was used to plot the lollipop graph.

### Drug sensitivity analysis

The gene expression data of 60 cancer cells prescribed by the National Cancer Institute (NCI) as mandatory screening for the development of new anti-cancer drugs and 163 drugs approved by the FDA were downloaded from the CellMiner database (https://discover.nci.nih.gov/cellminer/loadDownload.do). The IC_50_ of drugs was calculated between high and low OSBPL3 expression groups. Then, spearman correlation between OSBPL3 and drugs was analyzed. Finally, the ggpubr package (RRID:SCR_021139) was used to draw scatter plot.

### Immunohistochemistry (IHC)

As described in our previous publications, sections of CRC tissues were deparaffinized and rehydrated [23]. Antigen recovery was achieved by soaking the antigen in citrate buffer (pH 6.0) at 95 °C for 15 min before blocking endogenous peroxidase activity with 0.3% hydrogen peroxide at room temperature for 15 min. Primary anti-OSBPL3 antibody (1:50; Abcam Cat# ab58566, RRID: AB 2,283,138) was applied to sections after rinsing with phosphate-buffered saline (PBS) and blocking with 5% normal goat serum (Thermo Fisher Scientific Cat# 10000C, RRID: AB 2,532,979) for 30 min at room temperature. All sections were counterstained, dehydrated, and mounted with a coverslip at room temperature, using the peroxidase-antiperoxidase detection method. The proportion of positive colorectal cells was estimated using yellow particles in the cytoplasm and/or nucleus, and the strength of staining was graded as negative (–), weak positive ( +), medium positive (+ +), or strong positive (+ + +). The H-score was determined by multiplying the intensity score (which ranged from 0–3) by the percentage of positive cells (range 0–300) [24]. Two competent pathologists used a double-blind approach to determine the H-score.

### The quantitative real-time polymerase chain reaction (qRT-PCR) analysis

Eight pairs of CRC tissues were lysed with TRlzol Reagent (Ambion), and total RNA was isolated according to the instructions. RNA purity and concentration were tested with Nano drop. Reverse transcription of mRNA was performed using the surescript-first-strand-cDNA-synthesis-kit kit from Saville, the qRT-PCR reaction system consisted of 3 µl cDNA, 5 µl 2 × Universal Blue SYBR Green qPCR Master Mix, and 1 µl forward and reverse primers. The reaction was performed in a CFX96 real-time quantitative fluorescence PCR instrument (BIO-RAD) under the following conditions: pre-denaturation at 95 °C for 1 min, followed by 40 cycles that each involved incubation at 95 °C for 20 s, 55 °C for 20 s, and 72 °C for 30 s. The primers were synthesized by Tsingke Biotechnology, and the primer information was shown in Supplementary appendix: Supplementary Table 1. The internal control was GAPDH, three parallel experiments were set up for this experiment. Finally, the expression level of OSBPL3 between CRC tissue and para-cancerous samples was verified by qRT-PCR.

### Analytical statistics

All gene expression data was normalized using log^2^ transformational normalization. To compare the differences between normal and malignant tissues, two-group t-tests were used. We used Kaplan–Meier analyses, Cox proportional hazards models, and log-rank testing for all survival analyses in this investigation. The correlations between two variables were examined using Spearman's test or Pearson's test; a significant difference was defined as *P* < 0.05. All statistical studies were carried out using the R programming language.

## Results

### Differential expression analysis of OSBPL3

**Fig. 1:**
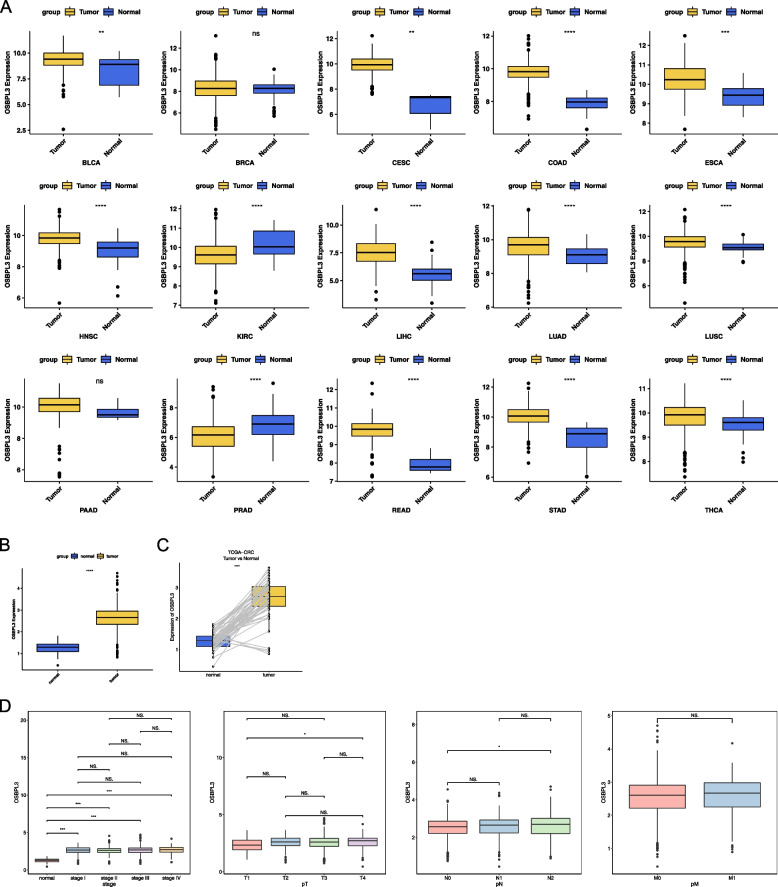
Differential expression analysis of OSBPL3. **A** Expression of OSBPL3 in GDC pan-cancer database. **B** Expression of OSBPL3 in CRC. **C** The pairing relationship of OSBPL3 in CRC. **D** Expression of OSBPL3 in clinical staging subgroups. nsP > 0.05, *P < 0.05, **P < 0.01,***P < 0.001

OSBPL3 was differentially expressed in 13 tumors (bladder cancer (BLCA), cervical cancer (CESC), colon adenocarcinoma (COAD), esophageal carcinoma (ESCA), head and neck squamous cell carcinoma (HNSC), kidney renal clear cell carcinoma (KIRC), liver hepatocellular carcinoma (LIHC), lung adenocarcinoma (LUAD), lung squamous cell carcinoma (LUSC), pancreatic adenocarcinoma (PAAD), rectum adenocarcinoma (READ), stomach adenocarcinoma (STAD), and thyroid carcinoma (THCA) with P < 0.01 (Fig. 1A), and most of them in tumor tissues were up-regulated. After merging COAD and READ data, OSBPL3 was also differentially expressed in CRC samples and normal samples (Fig. 1B). The paired relationship of CRC tumor and normal samples was used to draw the boxplots, and the results indicated that the genes were differentially expressed in the paired samples (Fig. 1C). In the different clinical stage subgroups, OSBPL3 was correlated with T stage and N stage, among which the expression of T4 was significantly higher than T1 and N2 was significantly higher than N0 (Fig. 1D).

### Multi-omics analysis of OSBPL3 in CRC

**Fig. 2:**
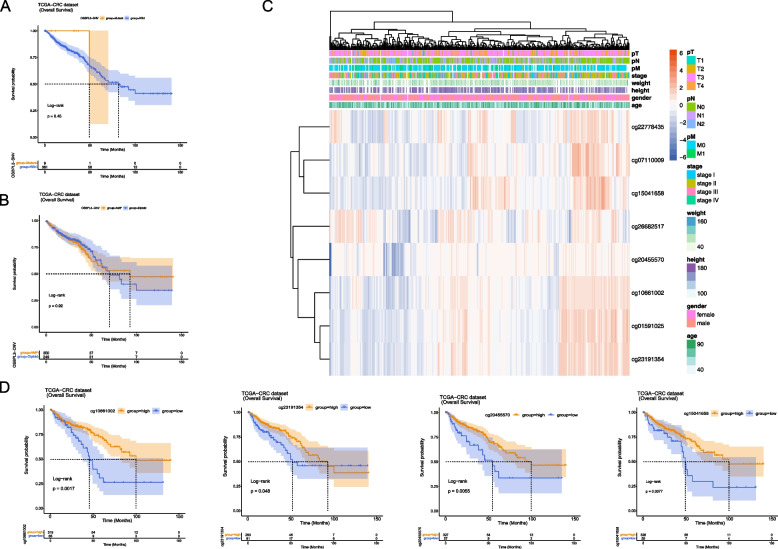
Multi-omics analysis of OSBPL3 in CRC. **A** Survival curve of OSBPL3 mutation and non-mutation group. **B** Survival curve of OSBPL3 amplification group and normal diploid group. **C** Distribution of methylation sites of OSBPL3 in different clinical subgroups. **D** Survival curve of methylation sites in OSBPL3

According to the SNV data, only 9 out of 381 samples were mutated. The OS of patients between mutant and wild groups was analyzed, and the results revealed that there was no effect of OSBPL3 mutations on patient survival (Fig. 2A). Based on the CNV data, among 616 samples, 361 samples were amplified, and only 3 samples had deletion. Then, the OS of patients between amplified and diploid groups was analyzed, and it was found that the CNV status of OSBPL3 did not affect the survival of patients (Fig. 2B). Distribution of methylation sites of OSBPL3 in different clinical subgroups, 8 differentially expressed methylation sites were obtained after annotation (Fig. 2C). The correlation analysis of the expression of methylation sites and OSBPL3 showed that 7 methylation sites were significantly negatively correlated with OSBPL3 (Table 1). Survival analysis between high and low methylation sites groups showed that 4 methylation sites (cg10661002, cg23191354, cg20455570, and cg15041658) were significantly related to the survival of patients (Fig. 2D).

**Table 1:** Correlation between methylation of OSBPL3 and its expression

**Gene**	**CpG**	**cor**	***p*-value**
OSBPL3	cg10661002	-0.414612401	0
OSBPL3	cg20455570	-0.385950873	3.52E-16
OSBPL3	cg07110009	-0.379293582	4.73E-15
OSBPL3	cg01591025	-0.3679306	2.16E-14
OSBPL3	cg23191354	-0.347803792	7.02E-13
OSBPL3	cg15041658	-0.23556164	1.66E-06
OSBPL3	cg22778435	-0.153972925	0.001856792
OSBPL3	cg26682517	-0.092084906	0.063475622

*Cor* correlation

### OSBPL3 correlated with survival of CRC patients

**Fig. 3:**
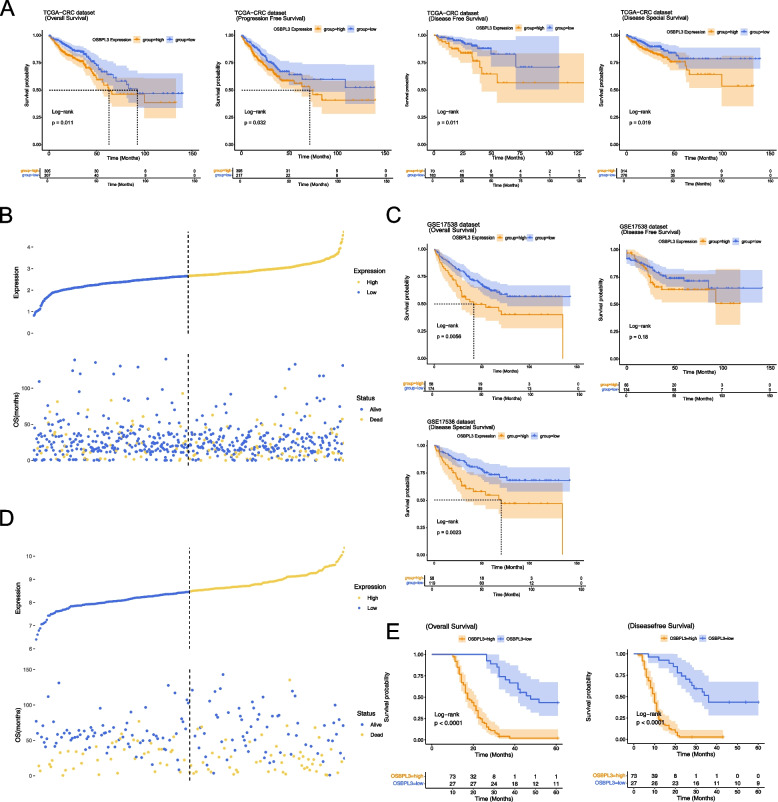
Survival analyses. **A** OS, PFS, DFS and DSS survival curves of OSBPL3 in TCGA-CRC dataset. **B** Expression curve of OSBPL3 in TCGA-CRC dataset and scatter plot of patients' survival status. **C** OS, DFS and DSS survival curves of OSBPL3 in GSE17538 dataset. **D** Expression curve of OSBPL3 in GSE17538 dataset and scatter plot of patients' survival status. **E** OS and DFS survival curves of OSBPL3 in clinical case data

The survival curves showed that OSBPL3 was significantly correlated with OS, DSS, DFS, and PFS of CRC patients, the higher OSBPL3 expression, the worse the prognosis of CRC patients (Figs. 3A). The expression curve of OSBPL3 and the scatter plot of patient status were drawn, and the results showed that more patients died in the OSBPL3 high expression group (Fig. 3B). In the GSE17538 validation set, OSBPL3 was significantly associated with OS, DSS of CRC patients (Fig. 3C). The expression profile of OSBPL3 and the scatter plot of patient status were shown, more patients died in the group with high OSBPL3 expression (Fig. 3D). In the self-test validation data, OSBPL3 had a significant correlation with OS and DFS in CRC patients (Fig. 3E).

### OSBPL3 and clinical application in patients with CRC

**Fig. 4:**
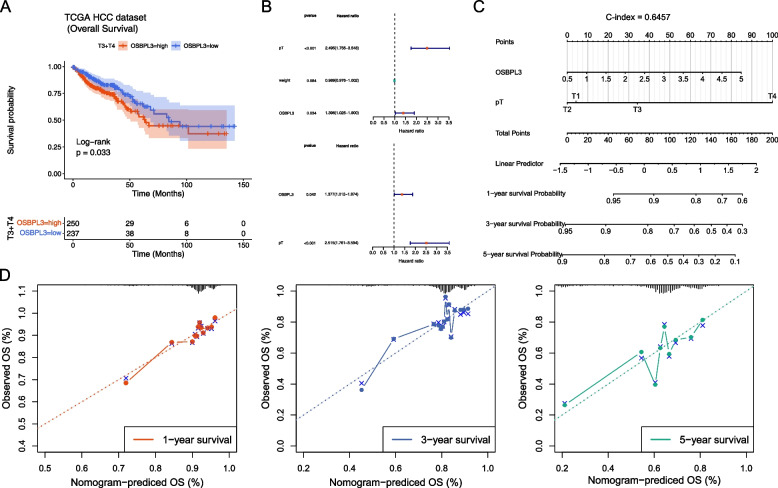
Construction of a nomogram. **A** Survival curve of patients with T3, T4 and weight less than 80 kg. **B** Univariate and multivariate Cox regression analyses of OS in CRC. **C** A nomogram for predicting survival in CRC patients. **D** Calibration diagram for predicting 1-, 3-, 5- year survival rate of patients

The clinical differences between patients with high and low OSBPL3 expression was analyzed, and it showed that OSBPL3 might be influenced by weight, vital, and pT-stage (Table 2). Stratified survival analysis of pT and weight showed that OSBPL3 remained significantly associated with survival in the subgroup of patients with T3/T4 and weight less than 80 kg (Fig. 4A). Moreover, univariate and multivariate Cox regression analyses showed that OSBPL3 and pT were independent prognostic factors (Fig. 4B). A nomogram for survival prediction in CRC patients was constructed using OSBPL3, pT and 1-, 3-, 5-year survival probability (Fig. 4C). The calibration curve was plotted based on the above nomogram, and indicating that the gene had a high accuracy in predicting the survival of patients at 1 and 3 years, but the prediction of 5-year survival was less satisfactory (Fig. 4D). Cox regression of 5 years OS in 100 CRC patients was shown in Tables 3 and 4. Furthermore, we explored the discrepancies of OSBPL3 in different sub-types of KRAS (NO and YES), BRAF (NO and YES), radiation therapy (YES and NO), therapy outcome (CR, PD, PR, and SD), stage (I, II, III, and IV) and MSI (MSI-H, MSI-L, and MSS) in the TCGA dataset. The results revealed that the expression of OSBPL3 in the KRAS mutant groups was significantly higher than that in the unmutated group (Supplementary Fig. 1).

**Table 2:** The clinical differences between CRC patients with high and low OSBPL3 expression

**Variables**	**OSBPL3**			***p*-value**
	**Total**	**high**	**low**	
**Age(year)**				
Mean (SD)	66.4 (± 12.7)	66.1 (± 12.6)	66.8 (± 12.8)	0.59
**Gender**				
Female	286 (46.7%)	134 (43.8%)	152 (49.7%)	0.17
Male	326 (53.3%)	172 (56.2%)	154 (50.3%)	
**Height**				
Mean (SD)	169.0 (± 11.8)	169.3 (± 12.8)	168.6 (± 10.5)	0.35
**Weight**				
Mean (SD)	80.8 (± 21.2)	83.7 (± 23.1)	77.2 (± 18.1)	0.029
**Vital**				
Alive	486 (79.4%)	232 (75.8%)	254 (83.0%)	0.036
Dead	126 (20.6%)	74 (24.2%)	52 (17.0%)	
**OS(Months)**				
Mean (SD)	27.3 (± 24.2)	25.9 (± 22.4)	28.7 (± 25.9)	0.22
**Stage**				
Stage I	103 (17.4%)	50 (16.9%)	53 (17.8%)	0.26
Stage II	226 (38.2%)	103 (34.9%)	123 (41.4%)	
Stage III	177 (29.9%)	93 (31.5%)	84 (28.3%)	
Stage IV	86 (14.5%)	49 (16.6%)	37 (12.5%)	
**pT**				
T1	19 (3.1%)	7 (2.3%)	12 (3.9%)	0.041
T2	104 (17.0%)	49 (16.0%)	55 (18.1%)	
T3	418 (68.5%)	205 (67.0%)	213 (70.1%)	
T4	69 (11.3%)	45 (14.7%)	24 (7.9%)	
**pN**				
N0	347 (57.0%)	163 (53.4%)	184 (60.5%)	0.17
N1	147 (24.1%)	77 (25.2%)	70 (23.0%)	
N2	115 (18.9%)	65 (21.3%)	50 (16.4%)	
**pM**				
M0	454 (84.2%)	220 (81.8%)	234 (86.7%)	0.13
M1	85 (15.8%)	49 (18.2%)	36 (13.3%)

**Table 3:** Univariate COX regression analysis of 5 years overall survival in 100 CRC patients

**Variables**	**Hazard Ratio**	**95%CI**	***p*-value**
Age(≥ 60 vs. < 60)	0.808	0.522–1.250	0.338
Gender(Male vs. Female)	1.138	0.739–1.752	0.557
Smoke(Smoker vs. Non_Smoker)	1.351	0.882–2.069	0.167
Drink(Alcoholic vs. Nonalcoholic)	0.830	0.540–1.276	0.395
CEA(≥ 5 vs. < 5)	9.232	5.002–17.037	0.000
Size(≥ 5 cm vs. < 5 cm)	0.724	0.472–1.110	0.139
TNMstage(3 + 4 vs. 1 + 2)	5.335	3.183–8.942	0.000
family(yes vs. no)	1.241	0.687–2.243	0.474
LynodeM(N1 + vs. N0)	6.883	3.371–12.697	0.000
OSBPL3(High vs. Low)	8.633	4.614–16.151	0.000

*CRC* colorectal cancer

**Table 4:** Multricariate cox of 5 years overall survival in 100 CRC patients

**Variables**	**Hazard Ratio**	**95%CI**	***p*-value**
CEA(≥ 5 vs. < 5)	1.593	0.495–5.128	0.435
TNMstage(3 + 4 vs. 1 + 2)	3.322	1.702–6.486	0.000
LynodeM(N1 + vs. N0)	1.310	0.409–4.194	0.650
OSBPL3(High vs. Low)	5.644	2.531–12.584	0.000

### KEGG and GO enrichment analysis of DEGs

**Fig. 5:**
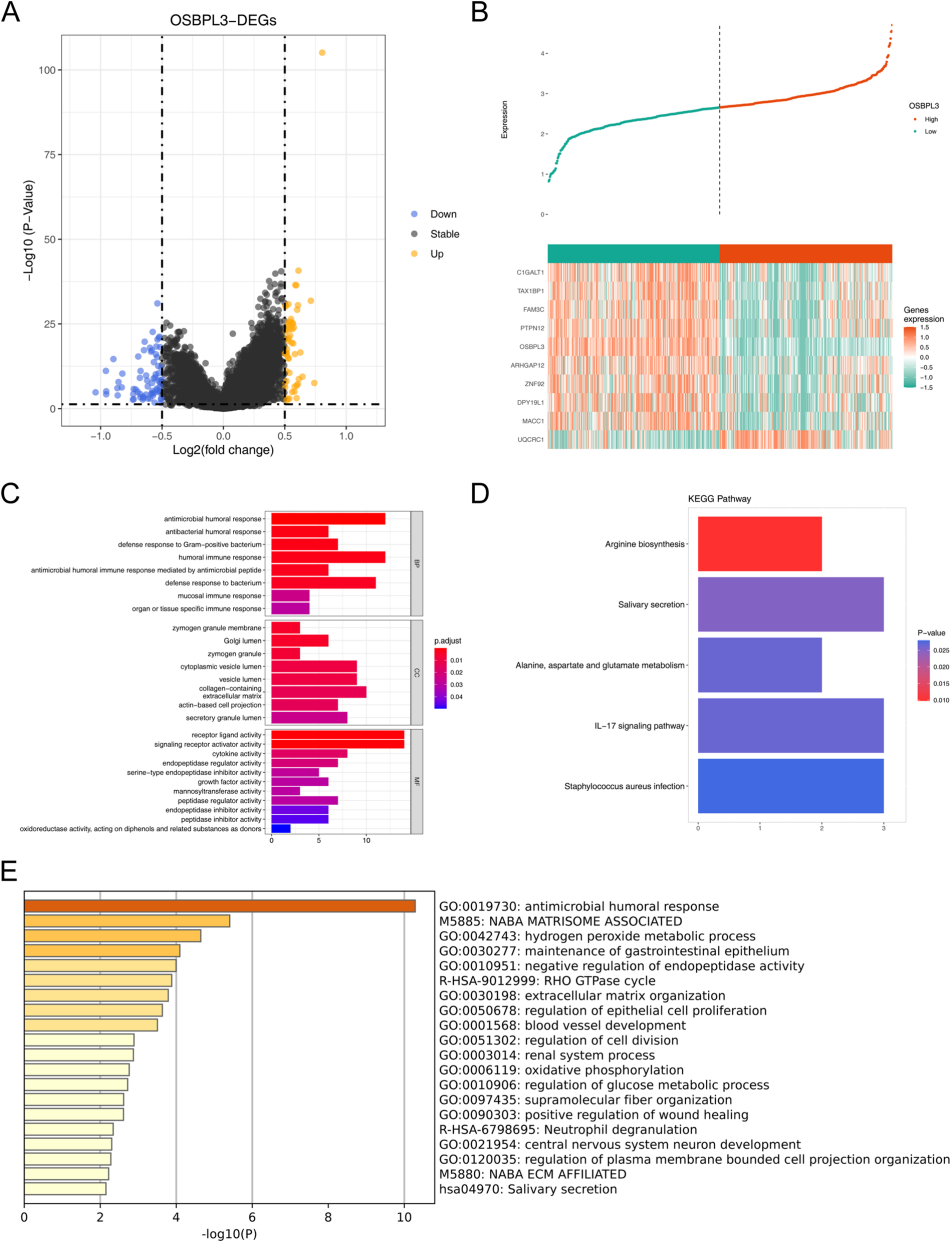
Identification of DEGs and their functional enrichment analysis. **A** Volcanic map of differentially expressed genes of OSBPL3. **B** Top10 differential expression gene heat map of OSBPL3. **C** GO of differentially expressed genes in OSBPL3.** D** KEGG of differentially expressed genes in OSBPL3. **E** Metascape of differentially expressed genes in OSBPL3

In total, 128 DEGs were obtained between high and low OSBPL3 expression groups, and top10 DEGs were showed in the heatmap (Fig. 5A-B, Supplementary appendix: Supplementary table 2). In terms of biological processes (BP), DEGs were significantly enriched in antibacterial humoral response, defense response to Gram-positive bacterium, and antimicrobial humoral responses, etc. In terms of molecular function (MF), DEGs were significantly enriched in signaling receptor activator activity receptor ligand activity, etc. In terms of cell composition (CC), DEGs were significantly enriched in zymogen granules, Golgi lumen, zymogen granule membrane, etc. (Fig. 5C). KEGG enrichment results showed that DEGs were significantly enriched in IL17 signaling pathway, alanine, aspartate and glutamate metabolism, salivary secretion, and arginine biosynthesis (Fig. 5D). Functional prediction of DEGs by Metascape showed that these genes might be significantly associated with antimicrobial humoral responses, hydrogen peroxide metabolic processes, negative regulation of endopeptidase activity, etc. (Fig. 5E). To obtain more pathways associated with CRC, we performed KEGG enrichment analysis after changing the screening threshold of DEGs. The results revealed that OSBPL3-related genes were associated with mTOR, FoxO, VEGF, MAPK and Ras signaling pathways and pathways linked with cancer and metabolism process (Supplementary Fig. 2A). Since previous studies [25] suggested a role for MAPK signaling pathway in CRC, we were interested in further mining its regulatory processes (Supplementary Fig. 2B).

### Analysis of immune cell correlation of OSBPL3

**Fig. 6:**
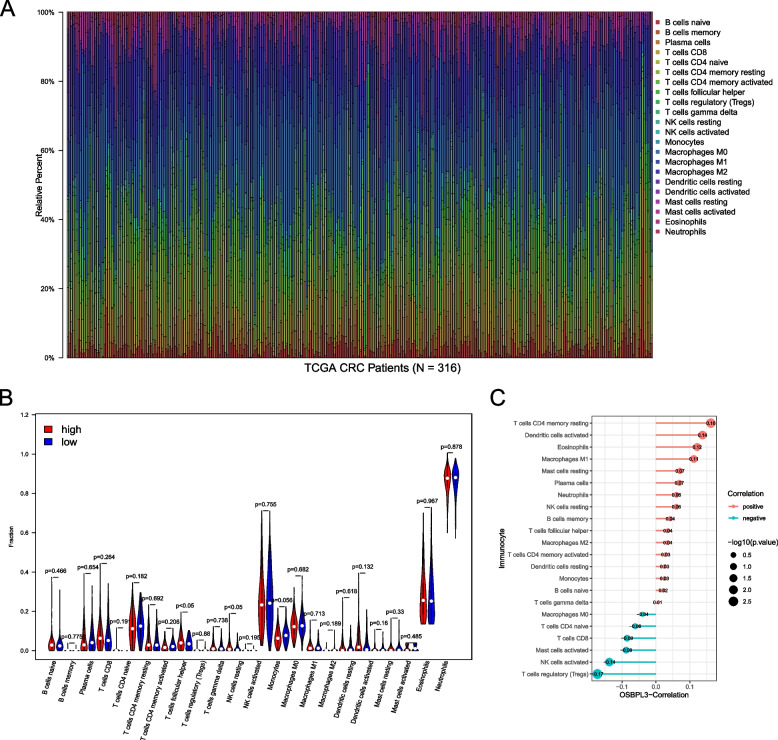
Correlation between OSBPL3 and immune cells. **A** CIBERSORT immune cell proportion stacking bar graph.** B** The difference of immune cells in high and low expression groups of OSBPL3. **C** Correlation between OSBPL3 and immune cells

The CIBERSORT algorithm was used to calculate the proportion of 22 immune cells in each sample, and 316 samples with *P* > 0.05 were obtained according to the corresponding statistical values (Fig. 6A). The differences of immune cells between high and low OSBPL3 expression groups showed that gamma delta T cells and follicular helper T cells were significantly different in two OSBPL3 expression groups (*P* < 0.05) (Fig. 6B). In addition, the correction analysis indicated that OSBPL3 was significantly correlated with resting memory CD4 T cell, activated Dendritic cells, activated NK cells, eosinophils, M1 macrophages, and regulatory T cells (Tregs) (Fig. 6C).

### Drug sensitivity analysis

**Fig. 7:**
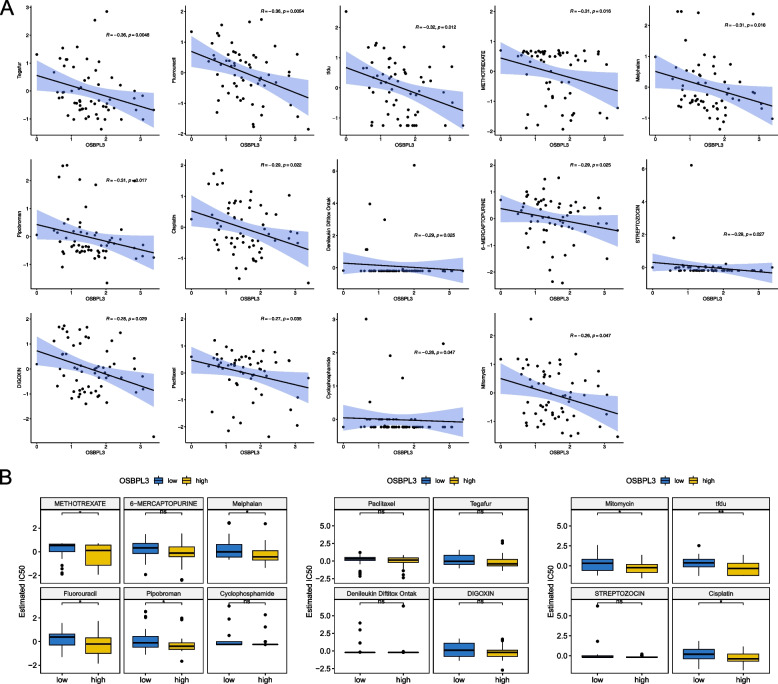
Drugs sensitivity. **A** Correlation between OSBPL3 and IC50 of FDA approved drugs.** B** IC50 of drugs between high and low expression groups of OSBPL3

To find the potential therapeutic drugs for patients in the high and low expression groups of OSBPL3, we performed a drug sensitivity analysis. A total of 14 drugs (tegafur, fluorouracil, tfdu, methotrexate, melphalan, pipobroman, cisplatin, denileukin diftitox ontak, 6-mercaptopurine, streptozocin, digoxin, paclitaxel, cyclophosphamide, and mitomycin) were screened (*P* < 0.05), and the IC_50_ of 14 drugs was negatively correlated with OSBPL3 (Cor < -0.2) (Fig. 7A, Supplementary appendix: Supplementary table 3). Then, the cell lines were divided into high and low OSBPL3 expression groups according to the median OSBPL3, and the IC_50_ of 14 drugs were compared between high and low OSBPL3 expression groups. The IC_50_ of methotrexate, melphalan, fluorouracil, pipobroman, mitomycin, tfdu and cisplatin was significantly different between high and low OSBPL3 expression groups (*P* < 0.05) (Fig. 7B). It could be seen that there was a negative correlation between OSBPL3 and multiple drugs, and there were differences in multiple drugs between high and low expression groups, indicating that OSBPL3 could be used to guide chemotherapy.

### Expression validation of OSBPL3

**Fig. 8:**
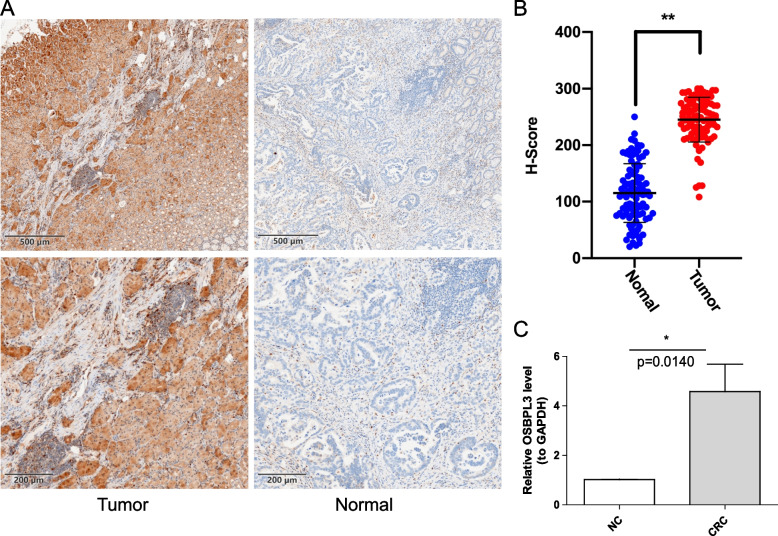
Validation the expression of OSBPL3. **A** IHC staining of CRC tissues (Tumor) and para-cancerous tissues(Normal) (*n* = 100; Scale bars, 500 mm and 200 mm).** B** The Scatter plot indicated the H-score of OSBPL3 IHC staining intensity.** C** OSBPL3 expression in CRC tissues (CRC) and matched para-cancerous tissues(NC) were measured by qRT-PCR (*n* = 8), and values were presented as mean ± standard deviation (SD). *P < 0.005, **P < 0.001)

We selected CRC tissue and para-cancerous samples from 100 CRC patients in Jiangmen Central Hospital for IHC analysis, and found that OSBPL3 was highly expressed in CRC samples (Fig. 8A-B). 8 pairs of CRC tissue and para-cancerous samples were collected and the expression level of OSBPL3 were verified by qRT-PCR. OSBPL3 was significantly upregulated in CRC samples (Fig. 8C). The results were consistent with the expression results of TCGA-CRC dataset.

## Discussion

CRC is the world`s third most common cancer and the second deadly cancer that has no effective diagnostic and prognostic signal for guiding the patient's detection and treatment [26]. Recently, many researchers have successfully revealed the important biological process of OSBPL3 in some cancers, such as gastric cancer [12], pancreatic ductal adenocarcinoma [27], breast cancer [28], etc. To our knowledge, the correlation between the expression of OSBPL3 and the prognosis of CRC has not yet been verified. Therefore, the focus of this study was on the potential pathways and prognostic value of OSBPL3 in CRC.

In the current study, we explored the expression of OSBPL3 in 15 types of tumor cells and their para-cancerous tissues, revealing that OSBPL3 has a higher expression in most tumors. As a cohort merged by COAD and READ, CRC cohort had a higher expression of OSBPL3 compared to its para-cancerous tissues and paired sample test also confirmed it. In fact, Hao etc. reported that OSBPL family genes were highly expressed in several tumor tissues, including CRC [29]. Chen etc. reported that OSBPL3 is upregulated in CRC [14]. But Xu’s study reported that colon tumor tissues had a lower expression of OSBPL3 than normal tissues and it is contrary to our findings [30]. Meanwhiles, our study revealed that OSBPL3 were associated with the T and N stage of CRC, it's similar to Hao's study. Furthermore, Zhang's research suggests that CRC samples with high OSBPL3 expression exhibit more KRAS mutations, which is consistent with our findings [31]. However, these results indicated the expression of OSBPL3 had changed in CRC.

Previous researches suggests that CNV, SNV and DNA methylation plays a role in the progression of tumors [32–35]. Therefore, we explored the connection of OSBPL3 with SNV, CNV or methylation for the first time. The results showed that OSBPL3 was not significantly associated with the SNV in CRC but correlated with CNV and methylationl. Our study indicated that the expression of OSBPL3 was significantly correlated with various CNV mutation patterns. In additional, we found 8 methylation sites which negatively correlated with OSBPL3 expression, and 4 methylation sites of them (cg10661002, cg23191354, cg20455570 and cg15041658) were correlated with OS. In fact, biomarkers of CRC such as INHBB, SMOC2, BDNF and TBRG4 were deregulated by methylation, and leading to the metastasis and poor prognosis of CRC [36], it indicated OSBPL3 might influence the prognosis of patients by regulating the methylation process in CRC.

Our study revealed the high expression of OSBPL3 predicted the worse OS, DFS and FPS. The GSE17538 cohort and self-test validation data validated the OSBPL3 was correlated the poor prognosis in CRC, indicating OSBPL3 had the prognostic value in CRC. At the same time, there were different overall survival curves between the OSBPL3 high expression and low expression cohorts in terms of tumor stage and weight difference. Furthermore, nomogram and COX regression analysis showed that the high expression of OSBPL3 suggested a poor prognosis in CRC patients. These results indicate that OSBPL3 is not only an independent risk factor for poor prognosis of CRC, but also a biomarker for prognosis evaluation of CRC.

In order to explore the biological functions of OSBPL3, we performed enrichment analysis and found the OSBPL3 related genes were mainly concentrated in metabolic signal pathway, such as “IL-17 signaling pathway”, “alanine, aspartic acid and glutamic acid metabolism”, “Regulation of glucose metabolic processes”, etc. As we know, the close connection between the immune and metabolic systems was irrebuttable. For example, IL-17 signaling pathway had the function on altering the tumor microenvironment (TME) by regulating the secretion of chemokines and cytokines, which could promote tumor progression [37]. In addition, a study found that PRDX2 may regulate the cell cycle progression and autophagy of CRC through the p38/FOXO pathway, and the cell cycle and FoxO pathways are also enriched by differentially expressed genes related to OSBPL3 [38]. Recent studies have shown that the mitogen-activated protein kinase (MAPK) signaling pathways may be activated by GFs and will further play key roles in CRC development [25]. The above indicates that OSBPL3 may participate in the occurrence and development of CRC through these pathways. Previous researches reports that the depletion of nutrients in TME could induce cancer cells to adapt by inducing nutrient scavenging mechanisms, which sustained cancer cell proliferation [39]. For further exploring the relationship of OSBPL3 with the TME, we analyzed the immune cells in the tumor microenvironment. The result showed OSBPL3 was intimate related with “T cells gamma delta” and “T cells follicular helper”. In CRC, γ/δ T cells can inhibit cancer progression through recognizing and attacking tumor cells, while it can also promote the cancer progression via secreting IL-17 [40]. Follicular helper T cells (Tfh) were helpful to shape germinal centers response [41]. A study showed Tfh was upregulated in tumors [42]. Thereby, we considered that OSBPL3 might regulate the TME mainly by upregulating the two cells, but further validation is still necessary.

Finally, OSBPL3 had a role in drug sensitivity. The low expression of OSBPL3 group had a higher IC50 for most chemotherapy drugs, including methotrexate, melphalan, fluorouracil, pipobroman, mitomycin, tfdu, and cisplatin, which means the high expression of OSBPL3 group had a higher sensitivity. In other words, patients in the high expression group could benefit more from these drugs. Methotrexate and melphalan were widely used anti-cancer agents [43, 44], and MiR-505 mediates methotrexate resistance in colorectal cancer by targeting RASSF8 [45]. Hyperthermic intraperitoneal chemotherap with Melphalan or Mitomycin-C had longer median progression-free survival in peritoneal carcinomatosis from CRC [44]. Fluorouracil could be used for colorectal cancer and liver metastasis [46]. However, our research findings suggest that OSBPL3 can serve as an important drug target with good clinical conversion prospects, which will provide a theoretical basis for the treatment of CRC.

However, there are still some limitations in this study report. Firstly, this single center study only included a small cohort. We plan to further investigate the role of OSBPL3 in the prognosis evaluation of CRC through a multicenter study. Secondly, there might have systematic bias because of the multiple information from different databases. Thirdly, the prognostic value and drug sensitivity of OSBPL3 needs to confirm in larger clinical trials.

## Conclusions

In conclusion, this is a unique and complete prognostic evaluation approach to investigate the link between OSBPL3 and CRC. The analysis and verification of OSBPL3 reveals the correlation with clinically prognostic value, DNA methylation, immune cell infiltration, and drug sensitivity. These are conducive to understanding the potential role of OSBPL3 in the prognosis evaluation of CRC.

### Supplementary Information

**Additional file 1: Supplementary Appendix: Supplementary Table 1.** The sequences of primer. **Supplementary Table 2.** 128 DEGs between high and low OSBPL3 expression groups. **Supplementary Table 3.** Correlations between OSBPL3 and drugs.

**Additional file 2: Supplementary Fig. 1.** Expression of OSBPL3 in different sub-types of KRAS, BRAF, radiation therapy, therapy outcome, stageand MSIin the TCGA dataset. NS: not significant; *p < 0.05, **p < 0.01, ***p < 0.001.

**Additional file 3: Supplementary Fig. 2.** Kyoto Encyclopedia of Genes and Genomespathways of differentially expressed genesbetween the high- and low-expression groups of OSBPL3.Top 20 KEGG pathways enriched in DEGs.The regulatory process of MAPK signaling pathway. The x-axis represents the number of genes, and the y-axis represents pathways.

## Data Availability

The datasets generated and/or analyzed during the current study are available in the (TCGA) database (https://portal.gdc.cancer.gov/), GSE17538 dataset in the Gene Expression Omnibus (GEO) database (https://www.ncbi.nlm.nih.gov/geo/), pan-cancer database of GDC (https://gdc.cancer.gov/about-data/publications/pancanatlas). The data sets used and/or analyzed in this study and the clinical validation data can be obtained from the corresponding authors according on reasonable requirements.

## Abbreviations

CRC: Colorectal cancer
OSBPL3: Oxysterol binding protein like 3
DEGs: Differentially expressed genes
PH: Pleckstrin homology
CNV: Copy number variation
SNV: Single nucleotide variation
TCGA: The Cancer Genome Atlas
GEO: Gene Expression Omnibus
KEGG: Kyoto Encyclopedia of Genes and Genomes
CIBERSORT: Cell Type Identification by Estimating Relative Subsets Of RNA Transcripts
NCI: National Cancer Institute
