# Supplementary material for: Prognostic and biological function value of OSBPL3 in colorectal cancer analyzed by multi-omic data analysis
Source: BMC Gastroenterol. 2023 Aug 7;23:270. doi: 10.1186/s12876-023-02824-1 (PMC10408063; doi:10.1186/s12876-023-02824-1)
Supplement: Supplementary file 1 — Additional file 1: Supplementary Appendix: Supplementary Table 1. The sequences of primer. Supplementary Table 2. 128 DEGs between high and low OSBPL3 expression groups. Supplementary Table 3. Correlations between OSBPL3 and drugs. [file 12876_2023_2824_MOESM1_ESM.docx]

**Supplementary Appendix**

**Supplementary table 1** The sequences of.primer.

| Gene name | Primer sequence | |
| --- | --- | --- |
|  | Forward primer | Reverse primer |
| OSBPL3 | GCAGTCTGTCTGCAAGTGGC | TGACAAGGGAGCCGAGAGTA |
| GAPDH | CCCATCACCATCTTCCAGG | CATCACGCCACAGTTTCCC |

**Supplementary table 2** 128 DEGs between high and low OSBPL3 expression groups.

|  | Symbol | logFC | AveExpr | t | P.Value | adj.P.Val | B |
| --- | --- | --- | --- | --- | --- | --- | --- |
| OSBPL3 | OSBPL3 | 0.80489403 | 2.631836494 | 26.90392977 | 8.20E-106 | 1.50E-101 | 229.2565407 |
| PTPN12 | PTPN12 | 0.611634517 | 3.856802561 | 14.56188968 | 1.85E-41 | 1.69E-37 | 83.31120541 |
| TAX1BP1 | TAX1BP1 | 0.585602123 | 4.256094015 | 13.64643018 | 3.30E-37 | 7.54E-34 | 73.65960483 |
| C1GALT1 | C1GALT1 | 0.593784532 | 2.752279303 | 13.63166842 | 3.85E-37 | 7.82E-34 | 73.50661801 |
| MACC1 | MACC1 | 0.712059306 | 2.888317299 | 12.60911099 | 1.44E-32 | 1.32E-29 | 63.13217362 |
| UQCRC1 | UQCRC1 | -0.537325135 | 6.68833246 | -12.42895399 | 8.77E-32 | 6.16E-29 | 61.35238496 |
| DPY19L1 | DPY19L1 | 0.530041164 | 3.19712032 | 12.37156229 | 1.55E-31 | 1.05E-28 | 60.78858854 |
| FAM3C | FAM3C | 0.60749308 | 3.098948622 | 12.30497731 | 3.01E-31 | 1.97E-28 | 60.13643087 |
| ZNF92 | ZNF92 | 0.509170797 | 2.05615035 | 12.26057299 | 4.68E-31 | 2.95E-28 | 59.70269109 |
| ARHGAP12 | ARHGAP12 | 0.504505141 | 3.162762098 | 12.22878735 | 6.41E-31 | 3.89E-28 | 59.3927894 |
| ARL4A | ARL4A | 0.600784581 | 3.293882827 | 11.99326277 | 6.50E-30 | 3.39E-27 | 57.11173534 |
| THOC2 | THOC2 | 0.550478562 | 3.045044101 | 11.34159257 | 3.41E-27 | 1.33E-24 | 50.94545367 |
| OSBPL8 | OSBPL8 | 0.545451931 | 2.559092124 | 11.12522108 | 2.60E-26 | 8.64E-24 | 48.94724262 |
| OXR1 | OXR1 | 0.528699954 | 2.663055319 | 11.10815904 | 3.05E-26 | 9.95E-24 | 48.7907497 |
| AKAP11 | AKAP11 | 0.564905388 | 2.903714211 | 11.04428418 | 5.52E-26 | 1.63E-23 | 48.20630256 |
| GLS | GLS | 0.564251475 | 3.362036985 | 10.99888726 | 8.41E-26 | 2.33E-23 | 47.79228789 |
| SLK | SLK | 0.526370413 | 3.368456098 | 10.93391957 | 1.53E-25 | 4.18E-23 | 47.20177015 |
| NT5C3A | NT5C3A | 0.52646678 | 3.831176411 | 10.76882544 | 6.98E-25 | 1.59E-22 | 45.71175437 |
| ANLN | ANLN | 0.574384929 | 3.340966003 | 10.71785381 | 1.11E-24 | 2.36E-22 | 45.25482425 |
| CBX3 | CBX3 | 0.562643821 | 5.642507271 | 10.59858595 | 3.27E-24 | 6.23E-22 | 44.19144418 |
| AHR | AHR | 0.541554357 | 3.587508591 | 10.59704041 | 3.32E-24 | 6.25E-22 | 44.17771783 |
| FAM91A1 | FAM91A1 | 0.513284505 | 3.355550742 | 10.53130119 | 6.00E-24 | 1.02E-21 | 43.59514578 |
| POLR2L | POLR2L | -0.575538327 | 6.276026346 | -10.38303681 | 2.26E-23 | 3.31E-21 | 42.29046563 |
| TMEM106B | TMEM106B | 0.502785735 | 2.553199023 | 10.35915911 | 2.80E-23 | 3.94E-21 | 42.08155322 |
| RB1CC1 | RB1CC1 | 0.543432301 | 2.924748665 | 10.10011466 | 2.75E-22 | 3.33E-20 | 39.83687992 |
| UQCRQ | UQCRQ | -0.520141522 | 6.745007843 | -10.01216123 | 5.91E-22 | 6.39E-20 | 39.08392493 |
| NDUFB7 | NDUFB7 | -0.526063727 | 7.408086777 | -9.929048899 | 1.21E-21 | 1.21E-19 | 38.37675016 |
| ZKSCAN1 | ZKSCAN1 | 0.532744897 | 3.889092486 | 9.902645677 | 1.53E-21 | 1.45E-19 | 38.1529816 |
| ARHGAP5 | ARHGAP5 | 0.515156418 | 2.897057582 | 9.847231138 | 2.46E-21 | 2.25E-19 | 37.68473974 |
| STARD10 | STARD10 | -0.536724804 | 5.671583485 | -9.790805139 | 3.99E-21 | 3.47E-19 | 37.20990705 |
| SKAP2 | SKAP2 | 0.531007812 | 3.122807237 | 9.683560241 | 9.94E-21 | 7.72E-19 | 36.31290393 |
| CHCHD10 | CHCHD10 | -0.641159102 | 5.356437157 | -9.612968101 | 1.81E-20 | 1.29E-18 | 35.72641334 |
| USP12 | USP12 | 0.555771523 | 3.520303522 | 9.530288877 | 3.62E-20 | 2.43E-18 | 35.04351751 |
| CD2AP | CD2AP | 0.568707881 | 3.901705383 | 9.471495322 | 5.92E-20 | 3.71E-18 | 34.56056065 |
| ANAPC11 | ANAPC11 | -0.545337325 | 4.527055803 | -9.227756619 | 4.45E-19 | 2.30E-17 | 32.58218105 |
| NAA38 | NAA38 | -0.51470986 | 4.152932808 | -9.171363946 | 7.05E-19 | 3.49E-17 | 32.1299749 |
| MRPL41 | MRPL41 | -0.597893765 | 5.361387666 | -9.122156878 | 1.05E-18 | 5.01E-17 | 31.73710234 |
| DEK | DEK | 0.516674463 | 5.437332517 | 9.018962621 | 2.43E-18 | 1.04E-16 | 30.91840912 |
| CDX1 | CDX1 | -0.646611335 | 6.79020138 | -8.914319385 | 5.61E-18 | 2.16E-16 | 30.09548889 |
| ENDOG | ENDOG | -0.581311375 | 3.133446638 | -8.905102929 | 6.04E-18 | 2.29E-16 | 30.02336308 |
| DPM3 | DPM3 | -0.589978455 | 5.383472731 | -8.791352541 | 1.49E-17 | 5.31E-16 | 29.13791428 |
| MAL2 | MAL2 | 0.509944795 | 6.324715628 | 8.738356017 | 2.27E-17 | 7.75E-16 | 28.72838841 |
| HSPH1 | HSPH1 | 0.62743392 | 4.559187643 | 8.717262658 | 2.67E-17 | 8.93E-16 | 28.56592589 |
| PCMTD2 | PCMTD2 | 0.515140676 | 3.540881018 | 8.692780765 | 3.24E-17 | 1.06E-15 | 28.37774731 |
| GOLGA4 | GOLGA4 | 0.500538199 | 3.122371657 | 8.619879144 | 5.73E-17 | 1.76E-15 | 27.81983643 |
| CYP4F3 | CYP4F3 | 0.58255492 | 1.988199395 | 8.576040412 | 8.05E-17 | 2.40E-15 | 27.48610976 |
| C12orf57 | C12orf57 | -0.549768033 | 4.503757431 | -8.561522506 | 9.01E-17 | 2.66E-15 | 27.37588453 |
| TWF1 | TWF1 | 0.517407241 | 4.232869988 | 8.482659754 | 1.66E-16 | 4.56E-15 | 26.7796928 |
| ITM2C | ITM2C | -0.693327126 | 7.269273308 | -8.363184582 | 4.13E-16 | 1.05E-14 | 25.88477224 |
| SDF2L1 | SDF2L1 | -0.539234916 | 5.159790046 | -8.309462463 | 6.21E-16 | 1.52E-14 | 25.48565005 |
| ESF1 | ESF1 | 0.52808025 | 2.936809941 | 8.233891728 | 1.10E-15 | 2.56E-14 | 24.92767325 |
| HMGB1 | HMGB1 | 0.532258411 | 5.218946868 | 8.176481209 | 1.69E-15 | 3.76E-14 | 24.50650507 |
| C4orf48 | C4orf48 | -0.895393038 | 4.429603724 | -8.130294329 | 2.38E-15 | 5.10E-14 | 24.16938887 |
| NUDT8 | NUDT8 | -0.566095706 | 3.396886905 | -7.848298126 | 1.89E-14 | 3.26E-13 | 22.14460628 |
| PRDX5 | PRDX5 | -0.530378473 | 8.833595519 | -7.475336472 | 2.68E-13 | 3.78E-12 | 19.55668787 |
| NRARP | NRARP | -0.506651904 | 4.695088786 | -7.327245612 | 7.45E-13 | 9.70E-12 | 18.5581163 |
| LRRC26 | LRRC26 | -0.956652636 | 1.944813306 | -6.98830293 | 7.29E-12 | 7.95E-11 | 16.33598115 |
| TMEM238 | TMEM238 | -0.674557309 | 4.088525855 | -6.983159531 | 7.54E-12 | 8.20E-11 | 16.30294753 |
| CLIC4 | CLIC4 | 0.510917391 | 4.313457125 | 6.976440533 | 7.89E-12 | 8.53E-11 | 16.25982577 |
| EPSTI1 | EPSTI1 | 0.538046325 | 3.353581375 | 6.789265184 | 2.67E-11 | 2.67E-10 | 15.07281904 |
| COMTD1 | COMTD1 | -0.527915477 | 4.374111927 | -6.736226864 | 3.76E-11 | 3.66E-10 | 14.74150157 |
| TFF3 | TFF3 | -0.82288465 | 8.891609892 | -6.703384364 | 4.63E-11 | 4.45E-10 | 14.5374638 |
| IFITM2 | IFITM2 | -0.551081081 | 6.098040098 | -6.626352932 | 7.55E-11 | 7.02E-10 | 14.062274 |
| EPHB3 | EPHB3 | -0.629109599 | 5.182920995 | -6.502696677 | 1.64E-10 | 1.44E-09 | 13.30941682 |
| DSG3 | DSG3 | 0.644039242 | 1.05116485 | 6.371567972 | 3.68E-10 | 3.07E-09 | 12.52455634 |
| CDC42EP5 | CDC42EP5 | -0.555473969 | 5.820192364 | -6.26404606 | 7.07E-10 | 5.63E-09 | 11.89143305 |
| TMEM160 | TMEM160 | -0.680086088 | 4.064784757 | -6.236139928 | 8.37E-10 | 6.58E-09 | 11.72865901 |
| CES3 | CES3 | -0.567540498 | 2.801146416 | -6.188608562 | 1.11E-09 | 8.57E-09 | 11.45288511 |
| RPL39 | RPL39 | 0.614417184 | 5.561006808 | 6.106011924 | 1.82E-09 | 1.35E-08 | 10.97809243 |
| SOCS1 | SOCS1 | -0.500008052 | 2.597505001 | -6.02927905 | 2.85E-09 | 2.05E-08 | 10.5420638 |
| GPT | GPT | -0.508707383 | 1.903923104 | -5.973887584 | 3.93E-09 | 2.75E-08 | 10.23034623 |
| HRCT1 | HRCT1 | -0.533524735 | 2.641559259 | -5.951193099 | 4.48E-09 | 3.11E-08 | 10.10337054 |
| RNF186 | RNF186 | -0.620802576 | 3.73602225 | -5.932775782 | 4.99E-09 | 3.42E-08 | 10.00064168 |
| HPGD | HPGD | 0.514108541 | 2.282869587 | 5.869921158 | 7.15E-09 | 4.79E-08 | 9.652185319 |
| CKB | CKB | -0.854026143 | 6.847489931 | -5.750949915 | 1.40E-08 | 8.95E-08 | 9.001709977 |
| GDF15 | GDF15 | -0.517135448 | 6.249614878 | -5.70897475 | 1.77E-08 | 1.12E-07 | 8.77505938 |
| SERPINB5 | SERPINB5 | 0.740397481 | 3.439545988 | 5.628356549 | 2.77E-08 | 1.70E-07 | 8.343936683 |
| RASD1 | RASD1 | -0.581457335 | 2.356375031 | -5.627295387 | 2.79E-08 | 1.71E-07 | 8.338298693 |
| AMN | AMN | -0.503319163 | 3.303723508 | -5.60911899 | 3.08E-08 | 1.87E-07 | 8.241875603 |
| CCDC88B | CCDC88B | -0.503891911 | 2.61697389 | -5.508438251 | 5.34E-08 | 3.12E-07 | 7.712879902 |
| ASPN | ASPN | 0.606648492 | 2.380608914 | 5.5060047 | 5.41E-08 | 3.16E-07 | 7.700200757 |
| CALD1 | CALD1 | 0.500666996 | 3.886813804 | 5.447380593 | 7.41E-08 | 4.23E-07 | 7.39629437 |
| SULF1 | SULF1 | 0.59810983 | 3.839824263 | 5.30948906 | 1.54E-07 | 8.33E-07 | 6.693117237 |
| S100P | S100P | -0.516198911 | 7.276381701 | -5.204808587 | 2.65E-07 | 1.37E-06 | 6.170274431 |
| SOD3 | SOD3 | -0.615965451 | 5.014337824 | -5.196800334 | 2.77E-07 | 1.43E-06 | 6.130667692 |
| C6orf15 | C6orf15 | 0.580811423 | 1.039616507 | 5.178470464 | 3.04E-07 | 1.56E-06 | 6.040222951 |
| C10orf99 | C10orf99 | -0.827835595 | 5.638480894 | -5.06698716 | 5.36E-07 | 2.64E-06 | 5.496443405 |
| ANTXR1 | ANTXR1 | 0.502741936 | 3.336888483 | 5.011758631 | 7.07E-07 | 3.42E-06 | 5.231083683 |
| LCN15 | LCN15 | -0.863174413 | 1.531123653 | -4.957662516 | 9.25E-07 | 4.40E-06 | 4.973761123 |
| TPSG1 | TPSG1 | -0.54227616 | 1.867829513 | -4.905399986 | 1.20E-06 | 5.57E-06 | 4.727607056 |
| DUOXA2 | DUOXA2 | -0.73047294 | 2.731668925 | -4.84348635 | 1.62E-06 | 7.38E-06 | 4.439116554 |
| WFDC2 | WFDC2 | -0.713377903 | 4.056083254 | -4.775918086 | 2.24E-06 | 9.99E-06 | 4.128150287 |
| FAM3D | FAM3D | -0.544096675 | 6.644965897 | -4.694893088 | 3.29E-06 | 1.43E-05 | 3.760599253 |
| SELENBP1 | SELENBP1 | -0.518098 | 5.933761445 | -4.629603324 | 4.48E-06 | 1.91E-05 | 3.468683649 |
| ATOH1 | ATOH1 | -0.579825406 | 2.361531461 | -4.556854647 | 6.27E-06 | 2.61E-05 | 3.147907805 |
| MUC2 | MUC2 | -0.953985229 | 4.583789909 | -4.512341526 | 7.69E-06 | 3.15E-05 | 2.953972742 |
| HBB | HBB | -0.530778703 | 3.013610889 | -4.491476287 | 8.46E-06 | 3.45E-05 | 2.86367967 |
| POSTN | POSTN | 0.579044432 | 4.251116497 | 4.475496723 | 9.09E-06 | 3.69E-05 | 2.794793843 |
| MUC5B | MUC5B | -0.697415711 | 3.009031088 | -4.430750441 | 1.11E-05 | 4.45E-05 | 2.603121868 |
| COL10A1 | COL10A1 | 0.560614066 | 2.127568 | 4.402263653 | 1.26E-05 | 5.00E-05 | 2.482038426 |
| PIGR | PIGR | -1.040746879 | 7.962338525 | -4.355950444 | 1.55E-05 | 6.06E-05 | 2.286748061 |
| SPDEF | SPDEF | -0.564416793 | 2.95344291 | -4.241979745 | 2.56E-05 | 9.62E-05 | 1.8144364 |
| SPINK1 | SPINK1 | -0.57959495 | 5.884287993 | -4.229659229 | 2.70E-05 | 0.000100956 | 1.764084641 |
| SMOC2 | SMOC2 | -0.510242079 | 3.513598587 | -4.196556225 | 3.11E-05 | 0.000115001 | 1.629482618 |
| PHGR1 | PHGR1 | -0.587320023 | 7.053698581 | -4.122355635 | 4.27E-05 | 0.000154071 | 1.331399531 |
| PI3 | PI3 | -0.677822888 | 6.065601822 | -4.07842935 | 5.13E-05 | 0.000182801 | 1.157305576 |
| LEFTY1 | LEFTY1 | -0.73376928 | 4.070179994 | -4.073590355 | 5.24E-05 | 0.000186213 | 1.138235017 |
| REG3A | REG3A | -0.953885212 | 3.108222491 | -4.044655832 | 5.91E-05 | 0.000207384 | 1.024651016 |
| LCN2 | LCN2 | -0.63013836 | 7.684050858 | -3.999997878 | 7.11E-05 | 0.000245737 | 0.850850155 |
| IGF2 | IGF2 | -0.86080454 | 3.440827856 | -3.86757281 | 0.0001217 | 0.000402821 | 0.346252189 |
| DUOX2 | DUOX2 | -0.573399419 | 3.146318318 | -3.841086702 | 0.000135263 | 0.000442505 | 0.247267801 |
| TFF1 | TFF1 | -0.652442629 | 6.402986936 | -3.652507639 | 0.000281943 | 0.000874747 | -0.438724089 |
| FCGBP | FCGBP | -0.676246978 | 4.196773154 | -3.521510496 | 0.000461031 | 0.001374367 | -0.895794869 |
| CXCL5 | CXCL5 | 0.500835344 | 2.110938758 | 3.445171965 | 0.00060975 | 0.001778536 | -1.154762225 |
| DMBT1 | DMBT1 | -0.650880515 | 3.701913696 | -3.393215427 | 0.000735383 | 0.002108298 | -1.327895489 |
| SFRP2 | SFRP2 | 0.595955189 | 3.411718292 | 3.36915633 | 0.000801379 | 0.002279619 | -1.407208949 |
| RETNLB | RETNLB | -0.505525915 | 2.433837654 | -3.339248094 | 0.0008911 | 0.002508155 | -1.505046648 |
| CEACAM7 | CEACAM7 | -0.596726471 | 4.622391434 | -3.296914931 | 0.001034128 | 0.00286672 | -1.642091658 |
| ZG16 | ZG16 | -0.614126255 | 2.373012742 | -3.284989486 | 0.001078106 | 0.002979141 | -1.680393353 |
| LYZ | LYZ | 0.536875473 | 6.995346525 | 3.284717456 | 0.001079129 | 0.002981066 | -1.681265486 |
| DEFA5 | DEFA5 | -0.730599472 | 2.993317961 | -3.164350916 | 0.001631402 | 0.004360988 | -2.060315539 |
| PPBP | PPBP | 0.52542762 | 1.547493424 | 3.143021539 | 0.001753017 | 0.004663566 | -2.126057249 |
| GUCA2A | GUCA2A | -0.508173529 | 3.643140421 | -3.129745606 | 0.001832876 | 0.004857626 | -2.166759204 |
| DEFA6 | DEFA6 | -0.657002672 | 3.003743395 | -3.093716377 | 0.002066793 | 0.005409307 | -2.276378346 |
| SPINK4 | SPINK4 | -0.736957543 | 5.097179158 | -3.067094586 | 0.00225694 | 0.005861628 | -2.356585168 |
| KRT23 | KRT23 | 0.514976545 | 3.493021396 | 2.95341053 | 0.003263477 | 0.008184899 | -2.691525766 |
| CLCA1 | CLCA1 | -0.559711337 | 3.563142629 | -2.405698437 | 0.016437251 | 0.034990229 | -4.132178368 |
| ITLN1 | ITLN1 | -0.5096329 | 3.496022489 | -2.321032006 | 0.020612815 | 0.042787633 | -4.32913328 |

**Supplementary table 3** Correlations between OSBPL3 and drugs.

| gene | drug | cor | p.value |
| --- | --- | --- | --- |
| OSBPL3 | Tegafur | -0.35972485 | 0.004758106 |
| OSBPL3 | Fluorouracil | -0.355138111 | 0.005362473 |
| OSBPL3 | tfdu | -0.323549159 | 0.011678603 |
| OSBPL3 | METHOTREXATE | -0.31058734 | 0.01572433 |
| OSBPL3 | Melphalan | -0.309351122 | 0.016166422 |
| OSBPL3 | Pipobroman | -0.30724767 | 0.01694311 |
| OSBPL3 | Cisplatin | -0.29473304 | 0.022253992 |
| OSBPL3 | Denileukin Diftitox Ontak | -0.289670394 | 0.024771607 |
| OSBPL3 | 6-MERCAPTOPURINE | -0.28912817 | 0.02505496 |
| OSBPL3 | STREPTOZOCIN | -0.285517322 | 0.027012436 |
| OSBPL3 | DIGOXIN | -0.282800217 | 0.028568801 |
| OSBPL3 | Paclitaxel | -0.273229966 | 0.034662129 |
| OSBPL3 | Cyclophosphamide | -0.25775227 | 0.046781165 |
| OSBPL3 | Mitomycin | -0.257161911 | 0.047304659 |
| OSBPL3 | Carboplatin | -0.2535227 | 0.050638591 |
| OSBPL3 | Thiotepa | -0.242528656 | 0.061890179 |
| OSBPL3 | Trametinib | 0.234244938 | 0.071633688 |
| OSBPL3 | Etoposide | -0.231096292 | 0.075643616 |
| OSBPL3 | Teniposide | -0.230450732 | 0.07648739 |
| OSBPL3 | Floxuridine | -0.229721488 | 0.077449514 |
| OSBPL3 | Olaparib | -0.228524367 | 0.079049721 |
| OSBPL3 | Eribulin mesilate | -0.222907636 | 0.086910284 |
| OSBPL3 | Cobimetinib (isomer 1) | 0.222667501 | 0.087259568 |
| OSBPL3 | Midostaurin | 0.222392043 | 0.087661588 |
| OSBPL3 | Nitrogen mustard | -0.220786441 | 0.090033933 |
| OSBPL3 | Triethylenemelamine | -0.217028846 | 0.095782601 |
| OSBPL3 | Vinblastine | -0.215221717 | 0.098647282 |
| OSBPL3 | ARRY-162 | 0.212480932 | 0.103118594 |
| OSBPL3 | Ixabepilone | -0.206142921 | 0.114058577 |
| OSBPL3 | Uracil mustard | -0.205651884 | 0.114941925 |
| OSBPL3 | 0ndrolone phenpropio0te | -0.203726607 | 0.118456021 |
| OSBPL3 | LEE-011 | 0.203271594 | 0.119298391 |
| OSBPL3 | Chlorambucil | -0.202556623 | 0.120631251 |
| OSBPL3 | Cytarabine | -0.19986661 | 0.125747854 |
| OSBPL3 | Oxaliplatin | -0.190974057 | 0.143836886 |
| OSBPL3 | Tamoxifen | -0.190713056 | 0.144395697 |
| OSBPL3 | Gemcitabine | -0.186481418 | 0.153683286 |
| OSBPL3 | Selumetinib | 0.186038121 | 0.154681246 |
| OSBPL3 | 6-THIOGUANINE | -0.180330149 | 0.167962405 |
| OSBPL3 | Epirubicin | -0.175265039 | 0.180429778 |
| OSBPL3 | Acetalax | -0.174187371 | 0.183166558 |
| OSBPL3 | Bosutinib | -0.173446705 | 0.185064798 |
| OSBPL3 | Topotecan | -0.172789419 | 0.186761177 |
| OSBPL3 | MITOXANTRONE | -0.172761938 | 0.186832346 |
| OSBPL3 | Po0tinib | 0.168836402 | 0.197199771 |
| OSBPL3 | Bortezomib | 0.167727235 | 0.200202019 |
| OSBPL3 | Vincristine | -0.162616401 | 0.214455668 |
| OSBPL3 | DECITABINE | -0.15895348 | 0.225099959 |
| OSBPL3 | Mitoxantrone | -0.158381728 | 0.226794029 |
| OSBPL3 | Neratinib | -0.154256577 | 0.239279178 |
| OSBPL3 | Fludarabine | -0.153671104 | 0.241088685 |
| OSBPL3 | ETHINYL ESTRADIOL | -0.151257008 | 0.248648877 |
| OSBPL3 | Zoledro0te | 0.150670017 | 0.250511289 |
| OSBPL3 | Irinotecan | -0.147529596 | 0.260636192 |
| OSBPL3 | Dasatinib | 0.146354341 | 0.264495196 |
| OSBPL3 | Raloxifene | -0.145050365 | 0.268821533 |
| OSBPL3 | Megestrol acetate | 0.143222897 | 0.274963928 |
| OSBPL3 | Hydroxyurea | -0.142281508 | 0.278164209 |
| OSBPL3 | PENTOSTATIN | -0.141054519 | 0.282372313 |
| OSBPL3 | Lenvatinib | 0.139662259 | 0.287197887 |
| OSBPL3 | Rapamycin | 0.138912308 | 0.289819545 |
| OSBPL3 | 7-Ethyl-10-hydroxycamptothecin | -0.13782728 | 0.293640245 |
| OSBPL3 | Parthenolide | -0.134843303 | 0.304316642 |
| OSBPL3 | Daunorubicin | -0.13447083 | 0.305666715 |
| OSBPL3 | BMN-673 | -0.13247144 | 0.312979747 |
| OSBPL3 | Azacitidine | -0.130791781 | 0.319209307 |
| OSBPL3 | Depsipeptide | 0.128623444 | 0.327367299 |
| OSBPL3 | LY-2835219 | 0.128322334 | 0.328510503 |
| OSBPL3 | Everolimus | -0.125873146 | 0.337902656 |
| OSBPL3 | Idarubicin | -0.124824588 | 0.341974507 |
| OSBPL3 | Arsenic trioxide | -0.124133332 | 0.344675493 |
| OSBPL3 | Carmustine | -0.123886675 | 0.345642474 |
| OSBPL3 | Pemetrexed | -0.121044744 | 0.356904986 |
| OSBPL3 | Ifosfamide | -0.12077118 | 0.358000861 |
| OSBPL3 | Actinomycin D | -0.120152831 | 0.360485506 |
| OSBPL3 | Nelarabine | -0.119716432 | 0.362245371 |
| OSBPL3 | Valrubicin | -0.117846475 | 0.369845515 |
| OSBPL3 | Cladribine | -0.117182833 | 0.372565811 |
| OSBPL3 | Vemurafenib | -0.116746333 | 0.374361606 |
| OSBPL3 | Axitinib | 0.114939975 | 0.381848373 |
| OSBPL3 | ABT-199 | 0.111937085 | 0.394490542 |
| OSBPL3 | Sunitinib | -0.111260855 | 0.39737111 |
| OSBPL3 | Ixazomib citrate | 0.110350003 | 0.401270565 |
| OSBPL3 | Estramustine | -0.1101848 | 0.401980204 |
| OSBPL3 | Sonidegib | -0.105760414 | 0.421256783 |
| OSBPL3 | Nelfi0vir | -0.105554246 | 0.422167721 |
| OSBPL3 | 6-Mercaptopurine | -0.10090596 | 0.443002329 |
| OSBPL3 | Fulvestrant | -0.098495557 | 0.454027579 |
| OSBPL3 | Gefitinib | -0.098074092 | 0.455970726 |
| OSBPL3 | Simvastatin | 0.096104654 | 0.465110856 |
| OSBPL3 | PF-06463922 | 0.094257325 | 0.473773645 |
| OSBPL3 | IPI-145 | 0.093096933 | 0.479258992 |
| OSBPL3 | Abiraterone | 0.091640062 | 0.486193354 |
| OSBPL3 | Acrichine | 0.091344886 | 0.487604733 |
| OSBPL3 | Allopurinol | -0.088205871 | 0.50274613 |
| OSBPL3 | VINORELBINE | -0.087727969 | 0.505072379 |
| OSBPL3 | Alectinib | 0.086639991 | 0.510388782 |
| OSBPL3 | Imatinib | -0.086093821 | 0.513068346 |
| OSBPL3 | Imiquimod | 0.084314682 | 0.521846182 |
| OSBPL3 | Calusterone | 0.080445717 | 0.54119082 |
| OSBPL3 | Lomustine | -0.079059622 | 0.548205079 |
| OSBPL3 | NMS-E628 | 0.078750233 | 0.549776673 |
| OSBPL3 | DAUNORUBICIN | -0.076837572 | 0.559540086 |
| OSBPL3 | Afatinib | 0.070602629 | 0.591923288 |
| OSBPL3 | Raltitrexed | -0.069888554 | 0.595684891 |
| OSBPL3 | Dexamethasone Decadron | -0.068726374 | 0.60182961 |
| OSBPL3 | Vinorelbine | -0.067974701 | 0.605818638 |
| OSBPL3 | TESTOLACTONE | -0.062570504 | 0.634830192 |
| OSBPL3 | AZACITIDINE | -0.061937313 | 0.638266544 |
| OSBPL3 | Homoharringtonine | -0.061331313 | 0.641562428 |
| OSBPL3 | LDK-378 | 0.058943683 | 0.654614727 |
| OSBPL3 | Idelalisib | -0.056354467 | 0.668886049 |
| OSBPL3 | brigatinib | 0.054993609 | 0.67643424 |
| OSBPL3 | Dexrazoxane | -0.054190752 | 0.680902355 |
| OSBPL3 | Isotretinoin | -0.052418012 | 0.690806655 |
| OSBPL3 | LOXO-101 | 0.050632958 | 0.700832108 |
| OSBPL3 | pralatrexate | -0.048730669 | 0.711572087 |
| OSBPL3 | Mithramycin | 0.046780541 | 0.722640304 |
| OSBPL3 | RAPAMYCIN | 0.046546757 | 0.723971028 |
| OSBPL3 | Docetaxel | -0.044813716 | 0.733860825 |
| OSBPL3 | Vorinostat | 0.044708726 | 0.73446136 |
| OSBPL3 | Celecoxib | 0.044250496 | 0.737084271 |
| OSBPL3 | Dromostanolone Propio0te | -0.043880004 | 0.739207153 |
| OSBPL3 | Cabozantinib | 0.042375825 | 0.747845694 |
| OSBPL3 | Erlotinib | -0.037735849 | 0.77468378 |
| OSBPL3 | Osimertinib | -0.03752189 | 0.775927962 |
| OSBPL3 | Panobinostat | 0.036034383 | 0.784593179 |
| OSBPL3 | Procarbazine | -0.035867308 | 0.785568093 |
| OSBPL3 | gilteritinib | 0.034318425 | 0.794621539 |
| OSBPL3 | ARSENIC TRIOXIDE | 0.032679376 | 0.804231423 |
| OSBPL3 | Doxorubicin | -0.030648679 | 0.816177493 |
| OSBPL3 | Crizotinib | 0.028926711 | 0.826340212 |
| OSBPL3 | Fluphe0zine | -0.02763781 | 0.83396573 |
| OSBPL3 | Pazopanib | -0.027427714 | 0.835210182 |
| OSBPL3 | Vandetanib | -0.026511415 | 0.840642317 |
| OSBPL3 | Palbociclib | 0.024537661 | 0.852368222 |
| OSBPL3 | Lapatinib | 0.024150965 | 0.854669362 |
| OSBPL3 | Irofulven | 0.023982215 | 0.855673933 |
| OSBPL3 | Ibrutinib | 0.023900176 | 0.856162398 |
| OSBPL3 | umbralisib | 0.023471958 | 0.858712877 |
| OSBPL3 | Bendamustine | -0.021779894 | 0.868804698 |
| OSBPL3 | Temsirolimus | 0.021365997 | 0.871276497 |
| OSBPL3 | Vismodegib | -0.020815675 | 0.874564928 |
| OSBPL3 | Asparagi0se | -0.019423681 | 0.882892035 |
| OSBPL3 | Dacomitinib | 0.01899254 | 0.885473784 |
| OSBPL3 | Encorafenib | 0.017703782 | 0.893198053 |
| OSBPL3 | JNJ-42756493 | -0.0162866 | 0.901703416 |
| OSBPL3 | Sulfatinib | -0.015616968 | 0.905726172 |
| OSBPL3 | DACARBAZINE | 0.015422664 | 0.906893875 |
| OSBPL3 | Mitotane | 0.014953426 | 0.909714663 |
| OSBPL3 | 6-Thioguanine | 0.012756201 | 0.922937278 |
| OSBPL3 | Copanlisib | -0.012725757 | 0.9230489 |
| OSBPL3 | TYROTHRICIN | -0.012588229 | 0.923949013 |
| OSBPL3 | Acalabrutinib | -0.010521351 | 0.936407607 |
| OSBPL3 | Pipamperone | -0.010086696 | 0.939029627 |
| OSBPL3 | Nilotinib | 0.009475776 | 0.942716029 |
| OSBPL3 | Bleomycin | -0.009086993 | 0.945062639 |
| OSBPL3 | Belinostat | 0.008558527 | 0.948253093 |
| OSBPL3 | tepotinib | 0.007475856 | 0.954791859 |
| OSBPL3 | Dabrafenib | -0.003538208 | 0.978595018 |
| OSBPL3 | Clofarabine | -0.002862145 | 0.982684279 |
| OSBPL3 | Carfilzomib | 2.80E-05 | 0.999830545 |
