# Supplementary figures and images for: Prognostic and biological function value of OSBPL3 in colorectal cancer analyzed by multi-omic data analysis
Source: BMC Gastroenterol. 2023 Aug 7;23:270. doi: 10.1186/s12876-023-02824-1 (PMC10408063; doi:10.1186/s12876-023-02824-1)

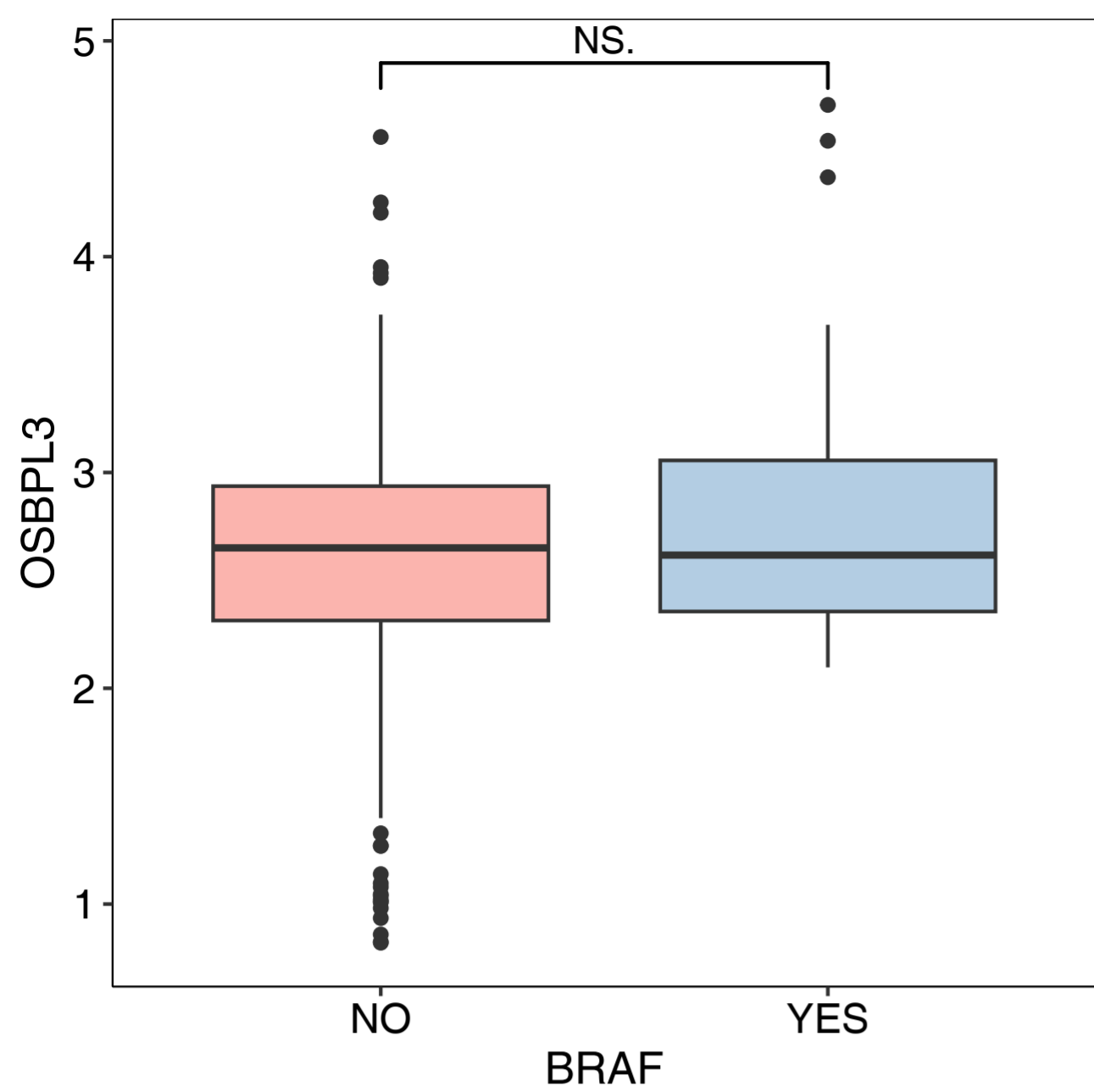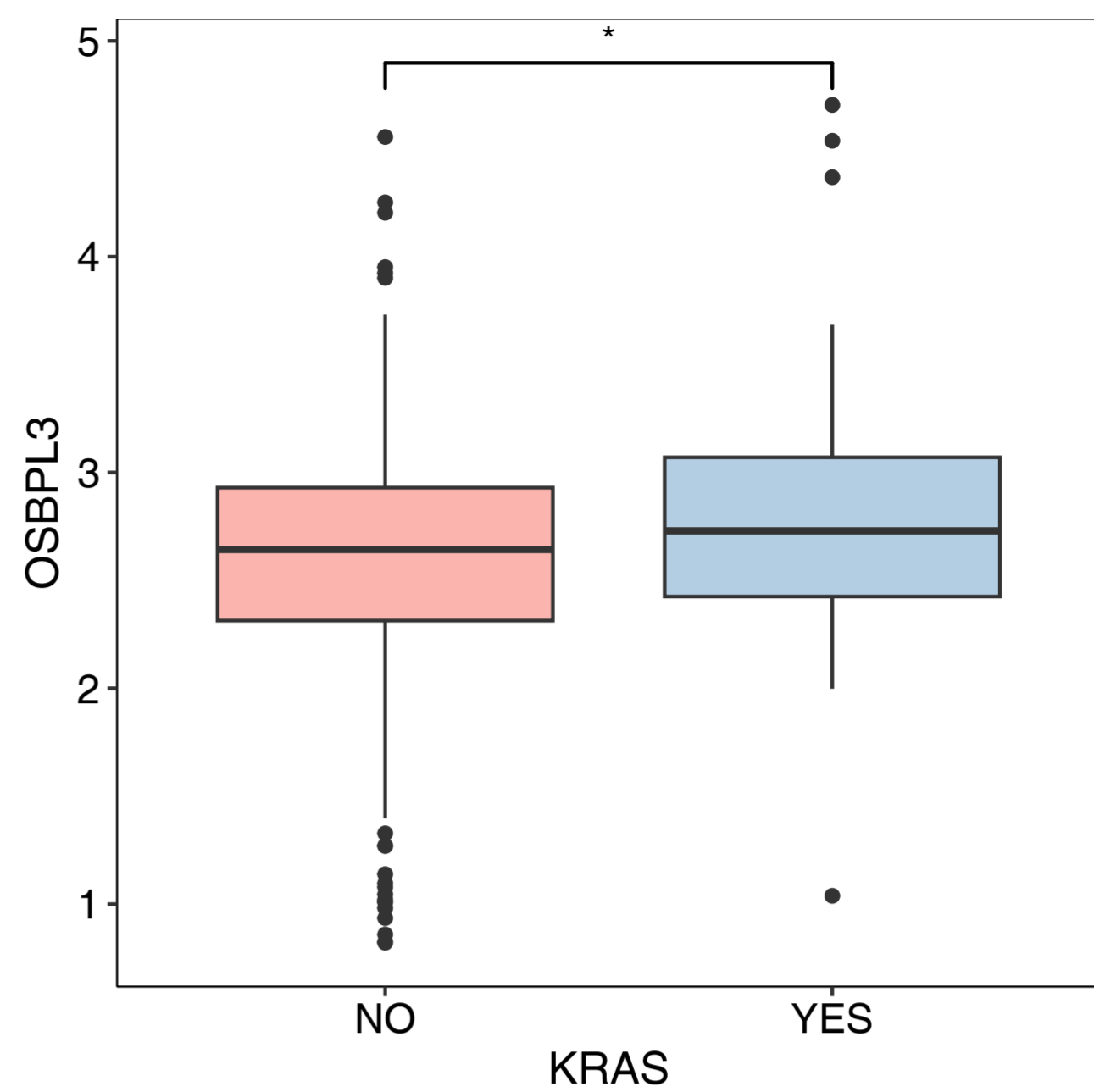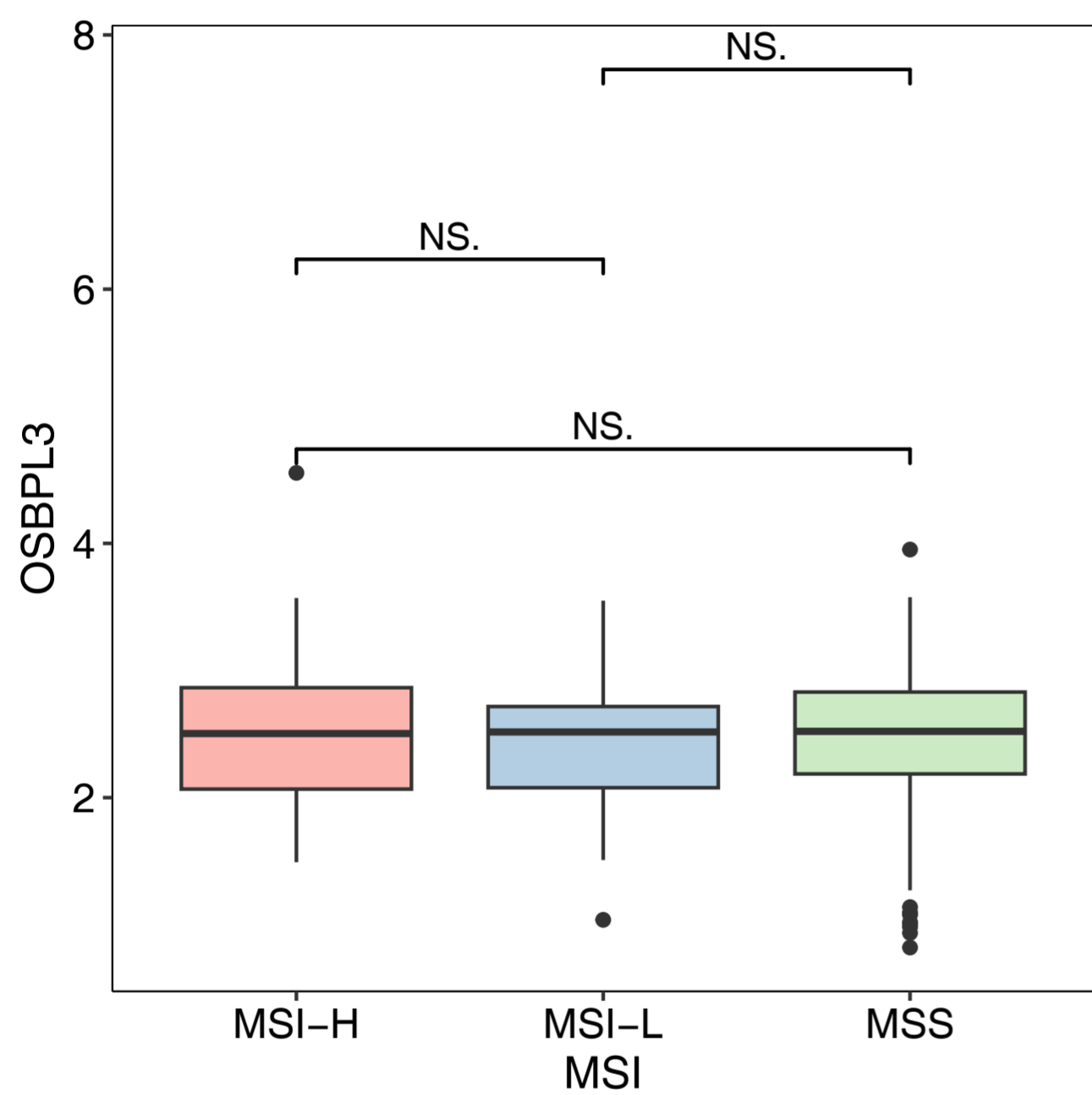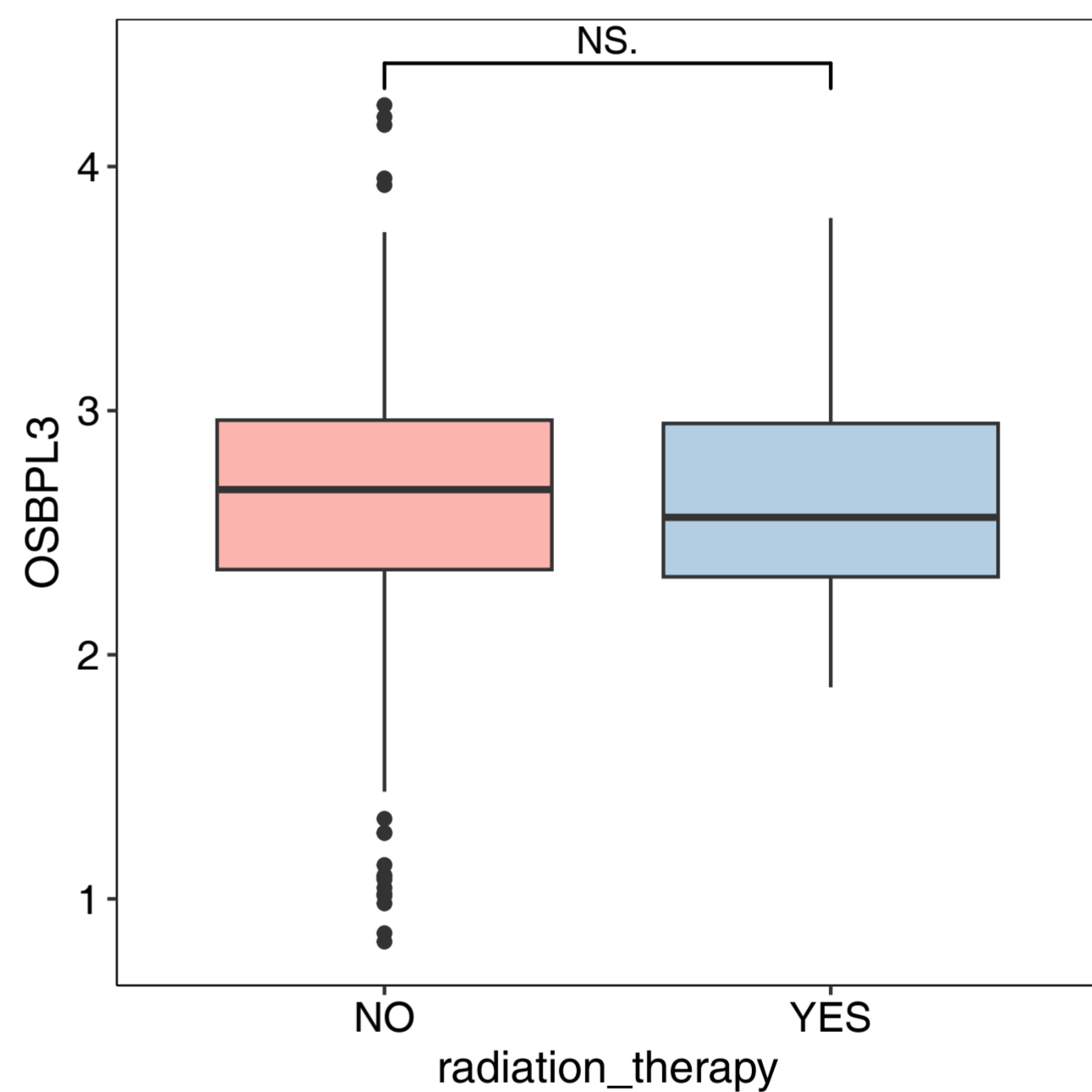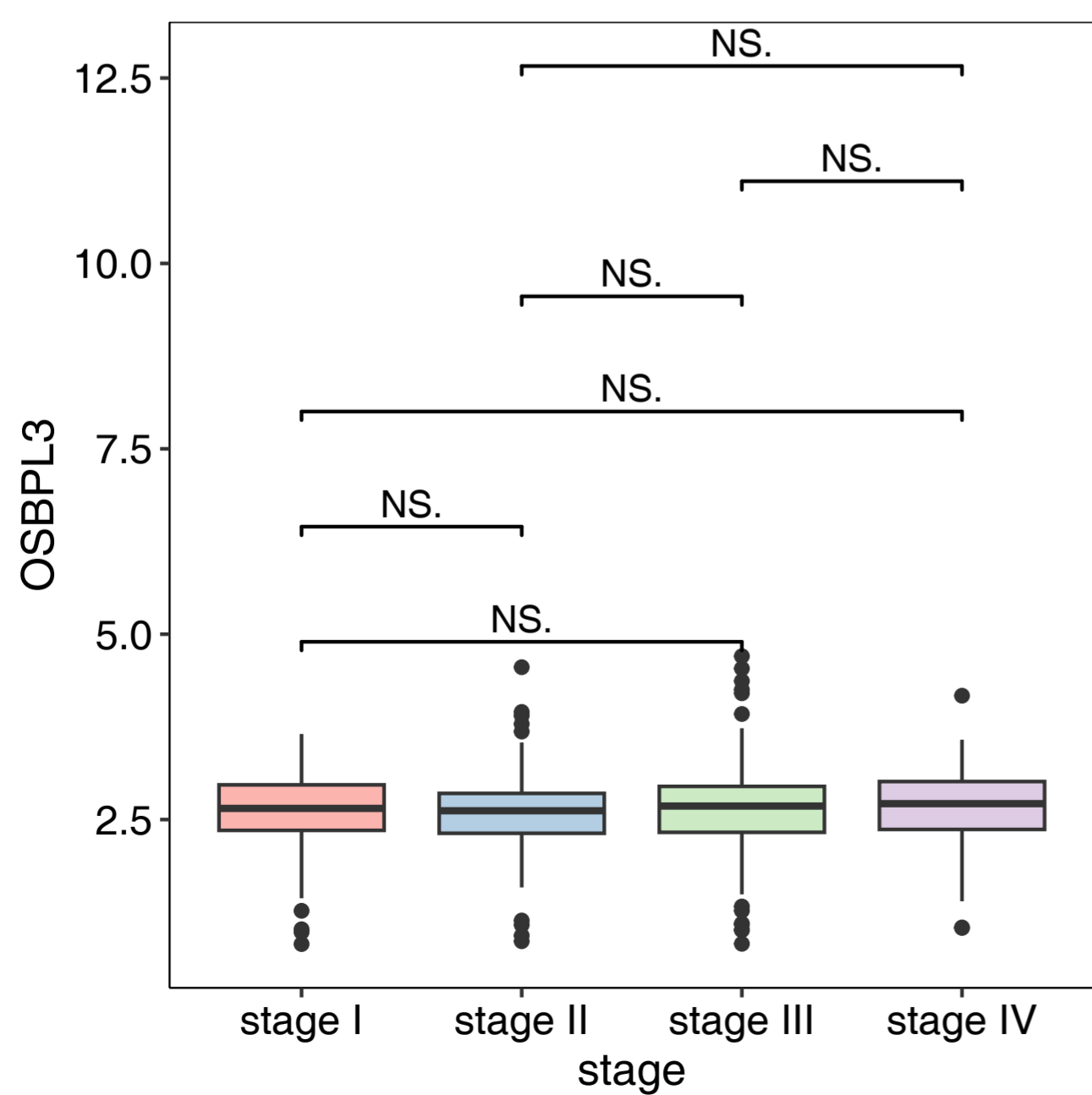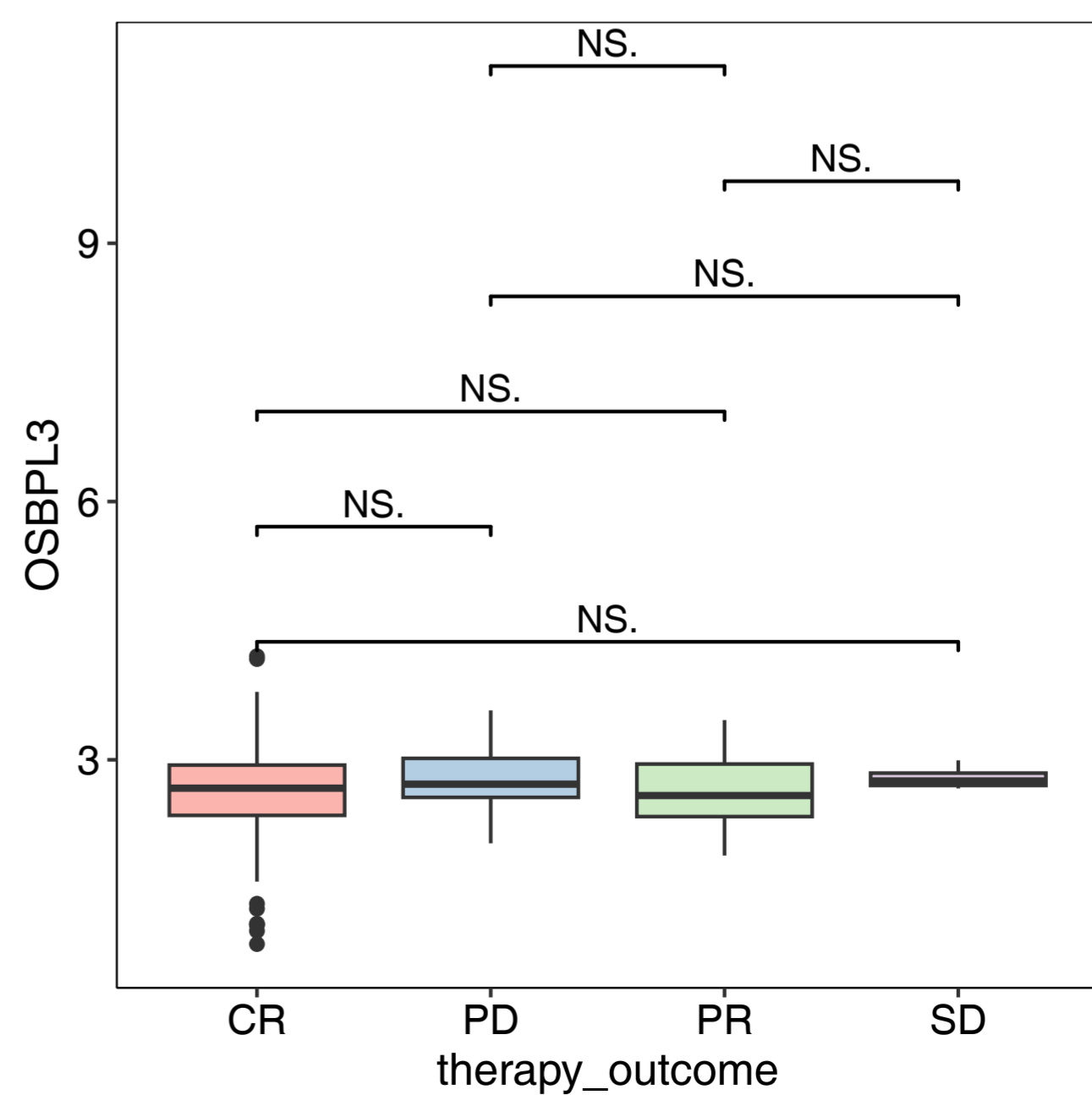

Supplement: Supplementary file 2 — Additional file 2: Supplementary Fig. 1. Expression of OSBPL3 in different sub-types of KRAS, BRAF, radiation therapy, therapy outcome, stageand MSIin the TCGA dataset. NS: not significant; *p < 0.05, **p < 0.01, ***p < 0.001. [file 12876_2023_2824_MOESM2_ESM.pdf]

A

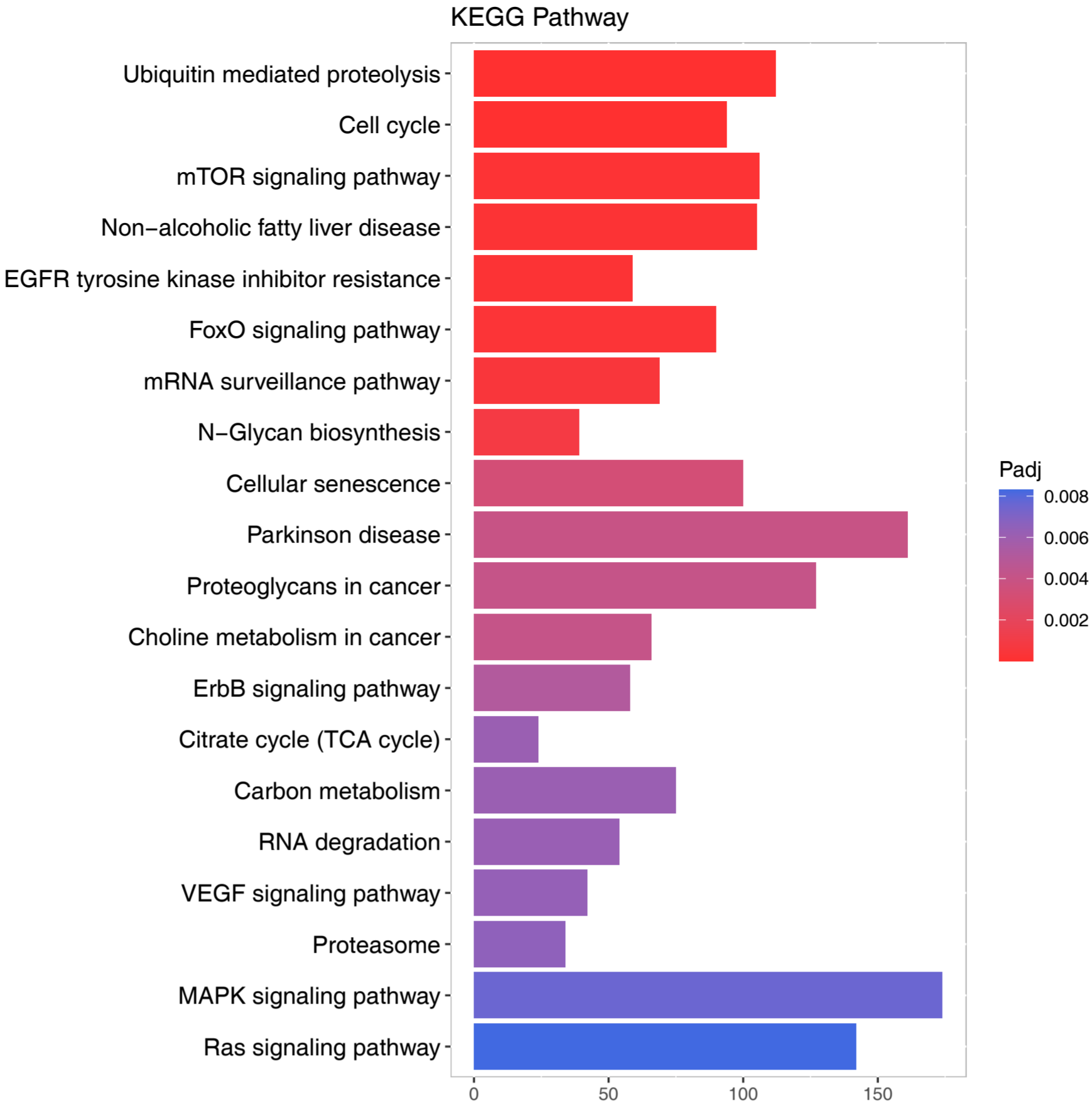

B

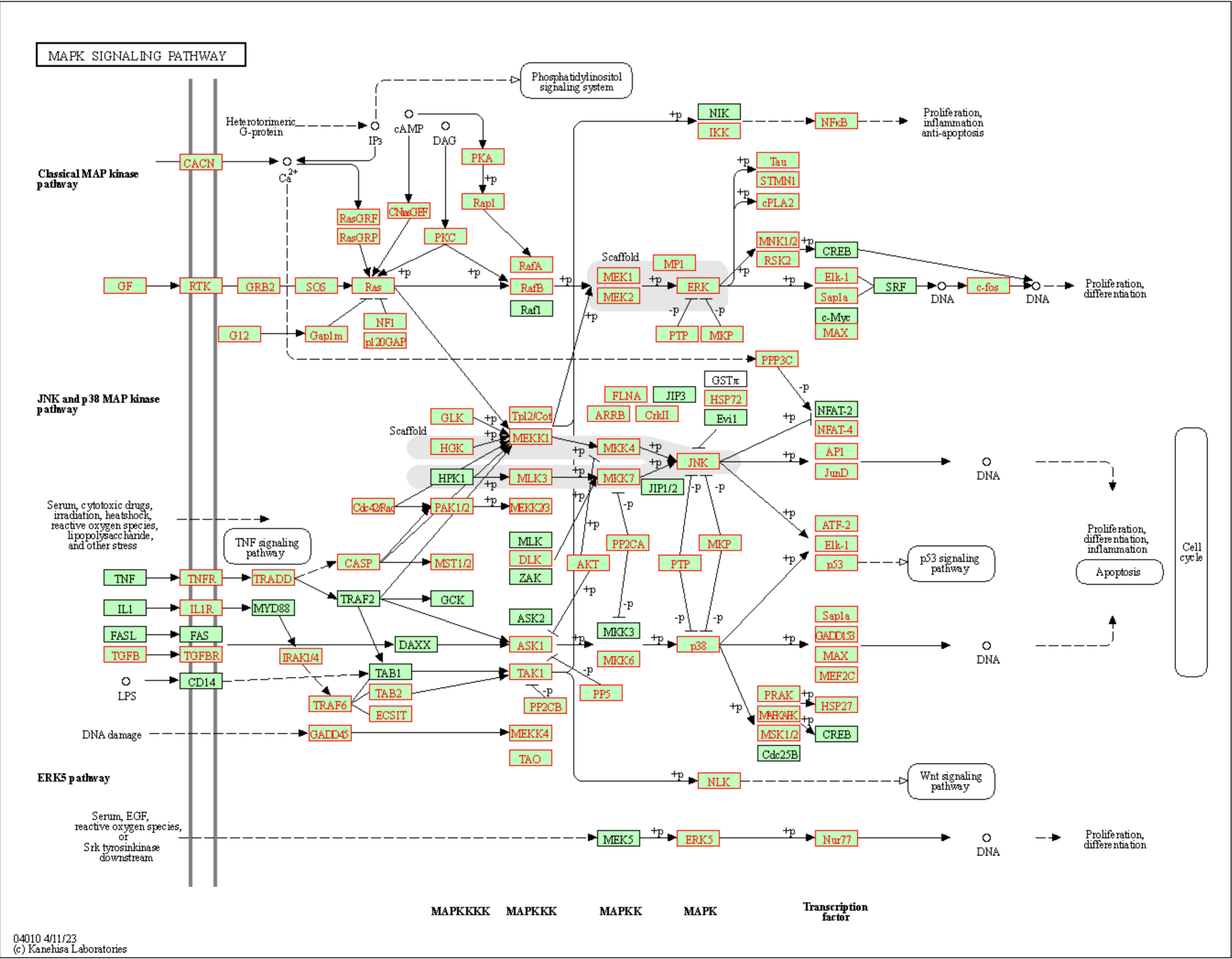

Supplement: Supplementary file 3 — Additional file 3: Supplementary Fig. 2. Kyoto Encyclopedia of Genes and Genomespathways of differentially expressed genesbetween the high- and low-expression groups of OSBPL3.Top 20 KEGG pathways enriched in DEGs.The regulatory process of MAPK signaling pathway. The x-axis represents the number of genes, and the y-axis represents pathways. [file 12876_2023_2824_MOESM3_ESM.pdf]
